# Near-Infrared Perylenecarboximide Fluorophores for Live-Cell Super-Resolution Imaging

**DOI:** 10.1021/jacs.3c13368

**Published:** 2024-03-05

**Authors:** Ze-Hua Wu, Xingfu Zhu, Qiqi Yang, Yulian Zagranyarski, Krishna Mishra, Hilmar Strickfaden, Ronald P. Wong, Thomas Basché, Kaloian Koynov, Mischa Bonn, Chen Li, Xiaomin Liu, Klaus Müllen

**Affiliations:** † Max Planck Institute for Polymer Research, Ackermannweg 10, 55128 Mainz, Germany; § Department of Chemistry, Johannes Gutenberg-University, 55099 Mainz, Germany; ∇ Katz Group Center, University of Alberta, Edmonton, AB T6G 2T9, Canada; # Institute of Molecular Biology (IMB), Ackermannweg 4, 55128 Mainz, Germany

## Abstract

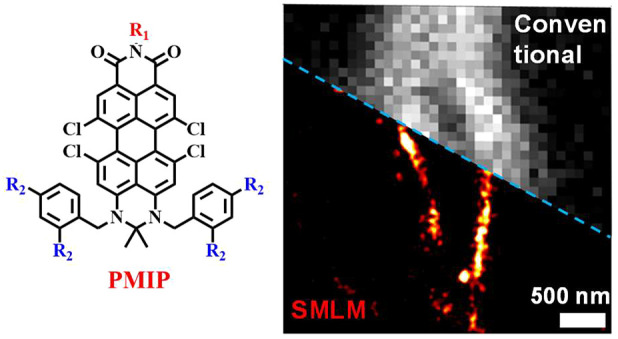

Organic near-infrared (NIR) photoblinking fluorophores are highly desirable for live-cell super-resolution imaging based on single-molecule localization microscopy (SMLM). Herein we introduce a novel small chromophore, **PMIP**, through the fusion of perylenecarboximide with 2,2-dimetheylpyrimidine. **PMIP** exhibits an emission maximum at 732 nm with a high fluorescence quantum yield of 60% in the wavelength range of 700–1000 nm and excellent photoblinking without any additives. With resorcinol-functionalized **PMIP** (**PMIP-OH**), NIR SMLM imaging of lysosomes is demonstrated for the first time in living mammalian cells under physiological conditions. Moreover, metabolically labeled nascent DNA is site-specifically detected using azido-functionalized **PMIP** (**PMIP-N**_**3**_) via click chemistry, thereby enabling the super-resolution imaging of nascent DNA in phosphate-buffered saline with a 9-fold improvement in spatial resolution. These results indicate the potential of **PMIP**-based NIR blinking fluorophores for biological applications of SMLM.

Near-infrared (NIR, 700–1000 nm) fluorescence imaging has received increasing attention in live-cell and *in vivo* imaging,^1^ due to lower phototoxicity, deeper tissue penetration, and higher signal-to-noise ratio compared to imaging in the visible region (400–700 nm).^2−5^ However, fluorescence imaging in the NIR region offers worse spatial resolution than in the visible region under the same conditions according to the wavelength-dependent Abbe diffraction limit.^6,7^ The theoretical spatial resolution of conventional microscopy in the NIR region exceeds 300 nm, making it impossible to resolve subcellular structures. Over the past decade, single-molecule localization microscopy (SMLM) has revolutionized conventional fluorescence microscopy by improving the spatial resolution from hundreds to tens of nanometers.^8,9^ This breakthrough enables the visualization of subcellular structures of dimensions below 200 nm.^10^ Although SMLM imaging in the NIR region holds promise for studying biological processes in living cells at a high spatial resolution, such as the dynamics of organelles, the availability of suitable fluorophores has remained limited.

The key requirement of SMLM is to achieve switching between fluorescent and nonfluorescent states of fluorophores, thereby enabling the localization precision of individual fluorophores within a few nanometers at different times and finally leading to the separation of fluorophores within a diffraction-limited area.^11,12^ Most reported organic fluorophores typically require special additives (e.g., oxygen scavenging systems and thiols) to facilitate the blinking performance.^13^ However, these additives are cytotoxic,^14^ thus limiting applications in living cells. On the other hand, the design of commonly used NIR organic fluorophores relies on extending the π-conjugation, which leads to poor photo/chemical stability^15^ and serious aggregation.^16^ Recently, a hybrid of single-walled carbon nanotubes (SWCNs) with photoswitching molecules (spiropyran-merocyanine) was reported to emit NIR fluorescence and exhibit intrinsic photoblinking in air.^17^ However, the large size (median length of 300 nm) and inherent hydrophobicity of SWCNs limit their applications in biological systems. In addition, SWCNs emit low photon numbers per blinking event, leading to poor localization precision.^17^ As such, developing NIR photoswitchable/blinking fluorophores for live-cell SMLM remains challenging.

Rylenecarboximide chromophores are a remarkable class of dyes with promising optical properties, such as outstanding thermal and photochemical stability, high red and deep-red fluorescence quantum yields (Φ_fl_), and biocompatibility, all of great importance for bioimaging.^18−21^ Perylene dicarboximide and terrylene dicarboximide have been reported to display photoblinking in single-molecule experiments.^22,23^ However, their utilization in live-cell super-resolution imaging, or biomolecule labeling and super-resolution imaging have not yet been reported in the NIR region. Herein, employing push–pull substitution, we develop a small-sized NIR fluorophore, named **PMIP**, based on the perylenecarboximide core fused with 2,2-dimetheylpyrimidine. **PMIP** exhibits an emission maximum (λ_em_) at 732 nm, high NIR Φ_fl_ of 60%, and excellent buffer-free blinking with a low on–off duty cycle of ∼10^–3^ and high photon numbers (per blinking event) of ∼1800. **PMIP** can be readily functionalized and used for SMLM bioimaging with significantly improved spatial resolution. Following cellular uptake of resorcinol-functionalized **PMIP** (**PMIP-OH**), NIR SMLM imaging of lysosomes is demonstrated for the first time in living cells under physiological conditions. In addition, super-resolution imaging of metabolically labeled nascent DNA is successfully achieved in phosphate-buffered saline (PBS) using azido-functionalized **PMIP** (**PMIP-N**_**3**_) via click reaction.

**PMIP** was synthesized through a two-step route commencing with **1** (Scheme 1). First, a metal-free coupling between **1** and benzylamine furnished **2a** in 70% yield. Subsequently, a six-membered pyrimidine ring at the peri-position of **2a** was formed to produce **PMIP** in 75% yield using acetone and a catalytic amount of trifluoroacetic acid. The chloro substituents in the bay region (1,6,7,12-positions) of perylene can induce a twisted skeleton to prevent π–π aggregation, while the phenyl rings of the benzyl units and the nitrogen center of the carboximide group allow further modification with functional groups for hydrophilicity and molecular targeting. **PMIP-OH** was obtained through methyl cleavage of **3** using BBr_3_ in 60% yield. In order to realize site-specific labeling, an azido function was introduced on **PMIP** to produce **PMIP-N**_**3**_. As depicted in Scheme 1, **4** was synthesized following the same protocol as for **PMIP**. Subsequent methyl cleavage with BBr_3_ afforded **5** in 85% yield. **6** was synthesized through nucleophilic substitution of **5** with 1,3-dibromopropane in 70% yield. Finally, a Menshutkin reaction with 3-azido-*N*,*N*-dimethylpropan-1-amine produced **PMIP-N**_**3**_ in 70% yield. The molecular structures were proven by nuclear magnetic resonance (NMR) spectroscopy and high-resolution matrix-assisted laser desorption/ionization time-of-flight mass spectrometry (HR MALDI-TOF MS; see the SI).

**Scheme 1:**
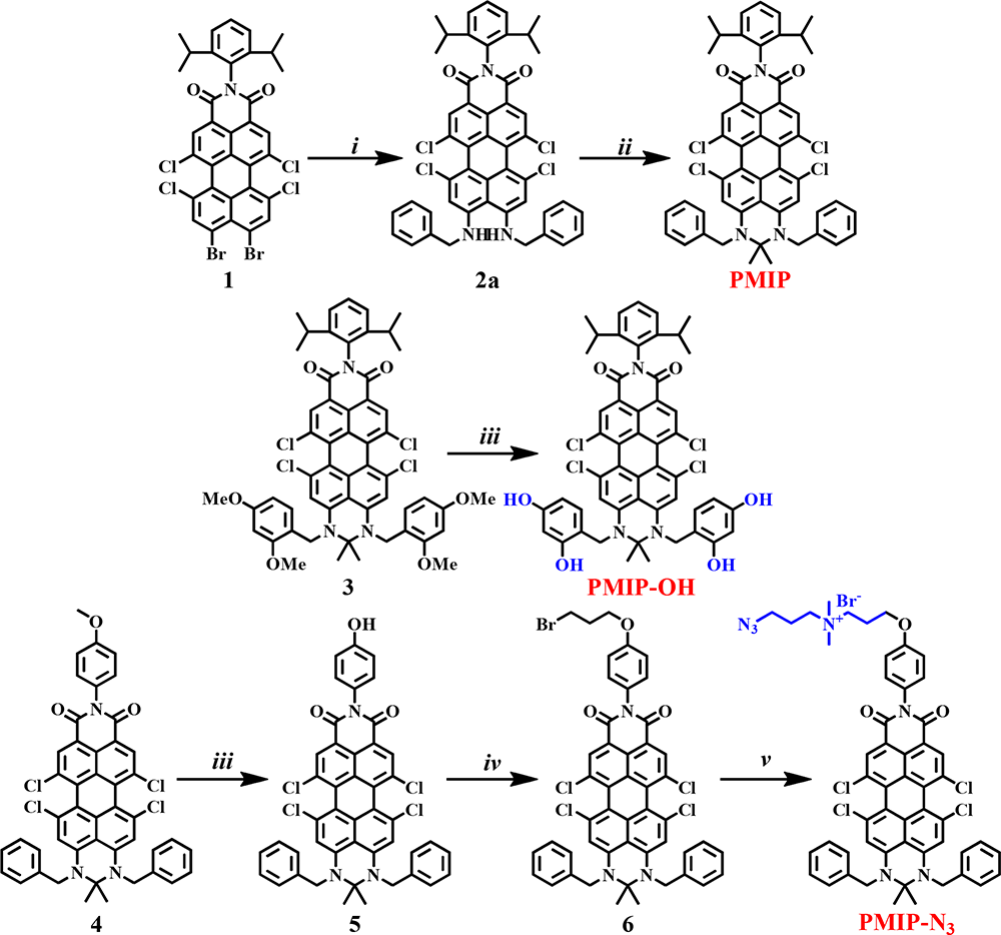
Synthesis of PMIP and Its Derivatives Reagents and conditions: (i) benzylamine, triethylamine, NMP, 90 °C, 30 min, 70% for **2a**; (ii) acetone, trifluoroacetic acid, 60 °C, 12 h, 75% for **PMIP**; (iii) BBr_3_, dichloromethane, 0 °C, 24 h, 60% for **PMIP-OH** and 85% for **5**; (iv) 1,3-dibromopropane, K_2_CO_3_, DMF, rt, 24 h, 70%; (v) (3-azidopropyl)dimethylamine, acetonitrile, 60 °C, 3 h, 70%.

As shown in Figure 1, **PMIP** exhibits absorption and emission maxima (λ_abs_ and λ_em_) at 660 and 732 nm, respectively, with a high molar extinction coefficient (ε) of 6 × 10^4^ M^–1^ cm^–1^ and a large Stokes shift of 72 nm. In comparison with the parent 3,4,9,10-tetrachloroperylene dicarboximide (λ_abs_ = 522 nm and λ_em_ = 550 nm),^24^**PMIP** realizes significant bathochromic shifts of 138 and 182 nm in absorbance and emission, respectively, resulting from intramolecular charge transfer (ICT). The ICT was also confirmed by cyclic voltammetry and density functional theory calculation (Figure S1). A high Φ_fl_ of 60% in the wavelength range of 700–1000 nm is achieved for **PMIP** in DMSO using Rhodamine 800 as a reference (Figure S2).^25^ The Φ_fl_ value of **PMIP** is twice as large as that of the guanidine analogue,^22^ enhancing imaging performance. **PMIP-OH** exhibits red-shifted λ_abs_ and λ_em_ at 700 and 750 nm, respectively, due to enhanced ICT compared with **PMIP** (Figure S3). This allows for excitation and emission both in the NIR region, which is beneficial for live-cell and *in vivo* imaging. **PMIP-N**_**3**_ demonstrates λ_abs_ and λ_em_ at 660 and 730 nm, respectively, with a Φ_fl_ of 58% (Table S1 and Figure S3). The water solubility of **PMIP-OH** and **PMIP-N**_**3**_ was measured with fluorescence correlation spectroscopy (FCS).^26^ Both compounds can be molecularly dissolved in DMSO (Figure S4), while the hydrodynamic radius (*R*_h_) of **PMIP-OH** and **PMIP-N**_**3**_ in water is 15 and 11 nm, respectively. Since the aggregates are sufficiently small, the impact on the following SMLM bioimaging is negligible. The absorption and emission changes of **PMIP** derivatives are negligible in buffer solutions with a pH range from 3 to 9 (Figures S5 and S6), suggesting them as potential fluorescent probes for use in cellular environments.

**Figure 1:**
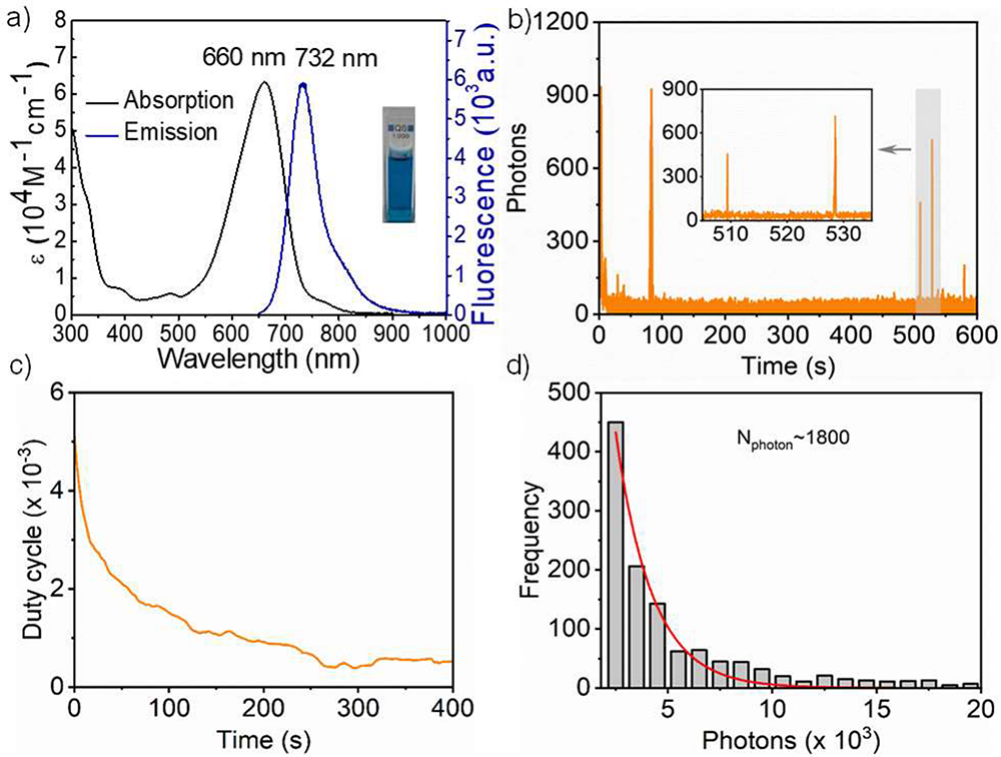
Photophysical properties of **PMIP**. (a) Absorption and emission spectrum in DMSO. (b) Typical single-molecule fluorescence time trace of **PMIP**. (c) On–off duty cycle of **PMIP** in air. (d) Histogram of detected photons per switching event and single-exponential fit of **PMIP** in air.

The single-molecule fluorescence blinking properties of **PMIP** were investigated in air without any additives (see the SI). **PMIP** exhibited excellent burst-blinking in the NIR domain (Figure 1b). We then measured the on–off duty cycle (fraction of time a molecule resides in its fluorescent state) and photon numbers (per blinking event), which are key parameters for achieving super-resolution imaging with SMLM.^13^**PMIP** displayed a low on–off duty cycle of ∼10^–3^ (Figure 1c), indicating a minimal occurrence of two individual molecules emitting fluorescence simultaneously in the diffraction-limited region, therefore contributing to high localization accuracy. Meanwhile, **PMIP** exhibited high photon numbers of ∼1800 (Figure 1d), corresponding to a theoretical resolution of ∼10 nm.^27^**PMIP-OH** and **PMIP-N**_**3**_ also showed high photon numbers of 1500 and 1900, respectively, with an on–off duty cycle of ∼10^–3^ (Figure S7). Taken together, **PMIP**-based fluorophores are suitable for achieving high-quality SMLM imaging.

For a proof of concept of live-cell SMLM imaging, U2OS cells were incubated with **PMIP-OH** (1 μM) in a standard cell culture medium for 30 min (see the SI). After cellular uptake, **PMIP-OH** selectively accumulated in lysosomes, as confirmed by a co-localization experiment with LysoTracker Green (Figure S8, Pearson’s correlation coefficient: 0.86). The lysosome is an organelle with a size of 200–300 nm that contains digestive enzymes to break down cellular wastes (e.g., proteins, nucleic acids, carbohydrates, and lipids). It thus plays an important role in mediating cellular metabolism and signaling.^28,29^ However, the natural acidic microenvironment of lysosomes (pH 4.5–5) has confined the use of pH-sensitive organic fluorophores.^30^ Here, the pH insensitivity of **PMIP-OH** allowed SMLM imaging of the lysosome. We therefore performed the live-cell SMLM imaging of lysosomes under physiological conditions in the NIR region. As displayed in Figure 2a, the conventional wide-field fluorescence image of lysosomes stained by **PMIP-OH** exhibited the typical diffraction-limited image. In contrast, the reconstructed SMLM image illustrated a much-improved resolution (Figure 2b). Furthermore, the real-time motion of individual lysosomes was monitored for 150 s with 30 s as intervals, enabling the observation of morphological changes with a spatial resolution in the range of tens of nanometers (Figure 2c). These results demonstrated the potential of **PMIP**-based blinking fluorophores for live-cell SMLM imaging in the NIR region.

**Figure 2:**
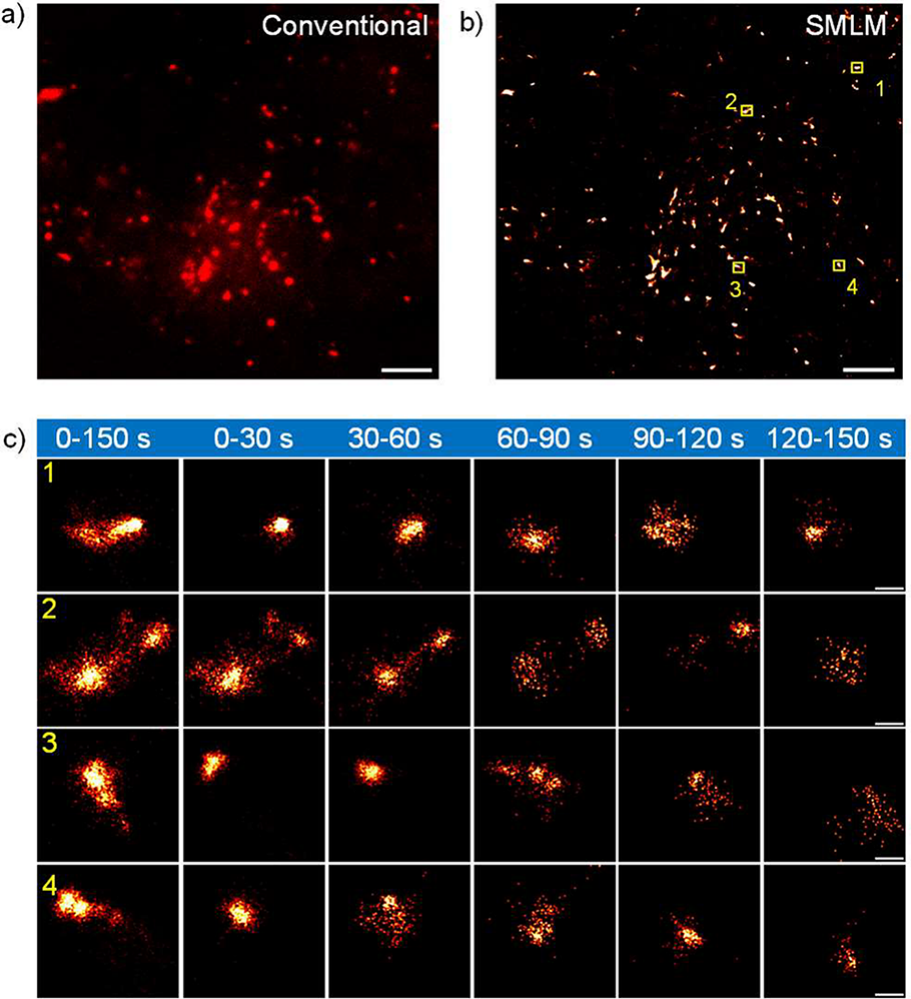
NIR live-cell SMLM imaging of lysosomes with **PMIP-OH**. (a) Conventional wide-field image of lysosomes labeled with **PMIP-OH** in living U2OS cells. (b) SMLM image of lysosomes labeled with **PMIP-OH** in living U2OS cells within 150 s. The four marked square areas (1, 2, 3, 4) represent four individual lysosomes. (c) Time sequence super-resolution images of lysosomes (yellow rectangle marked in b) with 30 s intervals. Scale bars: 2 μm in a and b, 200 nm in c.

In addition, we utilized **PMIP-OH** for the SMLM imaging of nanoscale crevices of a glass substrate in air (see the SI). With SMLM imaging, a full width at half-maximum (FWHM) of 74 nm was achieved for the nanoscale crevices, yielding a ∼10-fold improvement compared with the conventional fluorescence image (Figure S9).

Click chemistry is a powerful tool in the nucleic acid field.^31−33^ 5-Ethynyl-2′-deoxyuridine (EdU), a thymidine analog, is widely applied to metabolically incorporate into nascent DNA and can be subsequently site-specifically detected by azido-functionalized fluorophores via click chemistry, which contributes to the study of cell proliferation and differentiation,^34^ neurogenesis,^35^ carcinogenesis,^36^ and cell cycle dynamics.^37^ To achieve the super-resolution imaging of nascent DNA, **PMIP-N**_**3**_ was used to label the nascent DNA (see the SI). As illustrated in Figure 3a, U2OS cells were first incubated with (2′*S*)-2′-deoxy-2′-fluoro-5-ethynyluridine (F-ara-EdU) before washing with PBS. F-ara-EdU can be incorporated into the nascent DNA chain through covalent bonding during the replication process and possesses less toxicity than normally used EdU.^38^ Subsequently, the cells were lysed, and the DNA chains were extracted on a positively charged glass surface and then fixed in methanol/acetic acid solution. Finally, F-ara-EdU on the nascent DNA chains was detected by **PMIP-N**_**3**_ via click chemistry (for details see the SI). 4′,6-Diamidino-2-phenylindole (DAPI, blue commercial dye specifically labels DNA) was used for co-staining. The strong co-localization of **PMIP-N**_**3**_ and DAPI (Figure 3b, top panel, Pearson’s correlation coefficient: 0.86) indicated the successful labeling of **PMIP-N**_**3**_ to nascent DNA. In order to exclude the nonspecific adsorption of **PMIP-N**_**3**_, a control experiment was performed in which DNA was not pretreated with F-ara-EdU, but underwent the same click reaction with **PMIP-N**_**3**_ and was co-stained with DAPI. We observed the blue signal attributed to DAPI-labeled DNA, while no signal was detected in the NIR region (Figure 3b, bottom panel), illustrating negligible nonspecific targeting of DNA by **PMIP-N**_**3**_.

**Figure 3:**
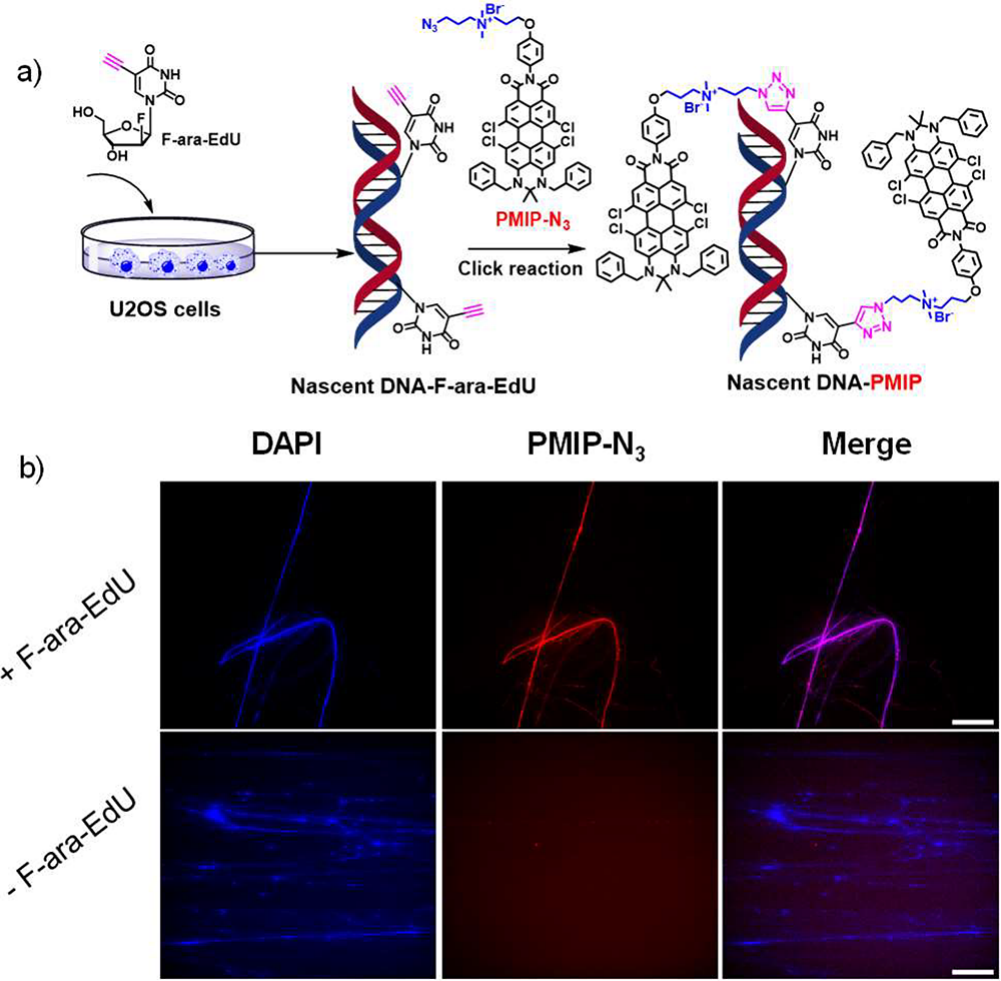
Labeling nascent DNA with **PMIP-N**_**3**_. (a) Diagram of nascent DNA incorporating with F-ara-EdU and subsequently detected with **PMIP-N_3_**. (b) Conventional wide-field images of nascent DNA metabolically labeled with (**+**)/without (**−**) F-ara-EdU and then performing the same click chemistry with **PMIP-N**_**3**_, scale bars: 50 μm.

NIR SMLM imaging of nascent DNA labeled by **PMIP-N**_**3**_ was performed in PBS solution without any additives. The SMLM image of nascent DNA chains displayed a remarkably high resolution (Figure 4a), featuring an FWHM of approximately 60 nm, which represents a 9-fold improvement when compared to conventional wide-field imaging (FWHM = 520 nm, Figure 4c). Moreover, SMLM imaging enabled the clear distinction of densely packed DNA chains with a higher signal-to-noise ratio, which is challenging in conventional fluorescence imaging (Figure 4b,d).

**Figure 4:**
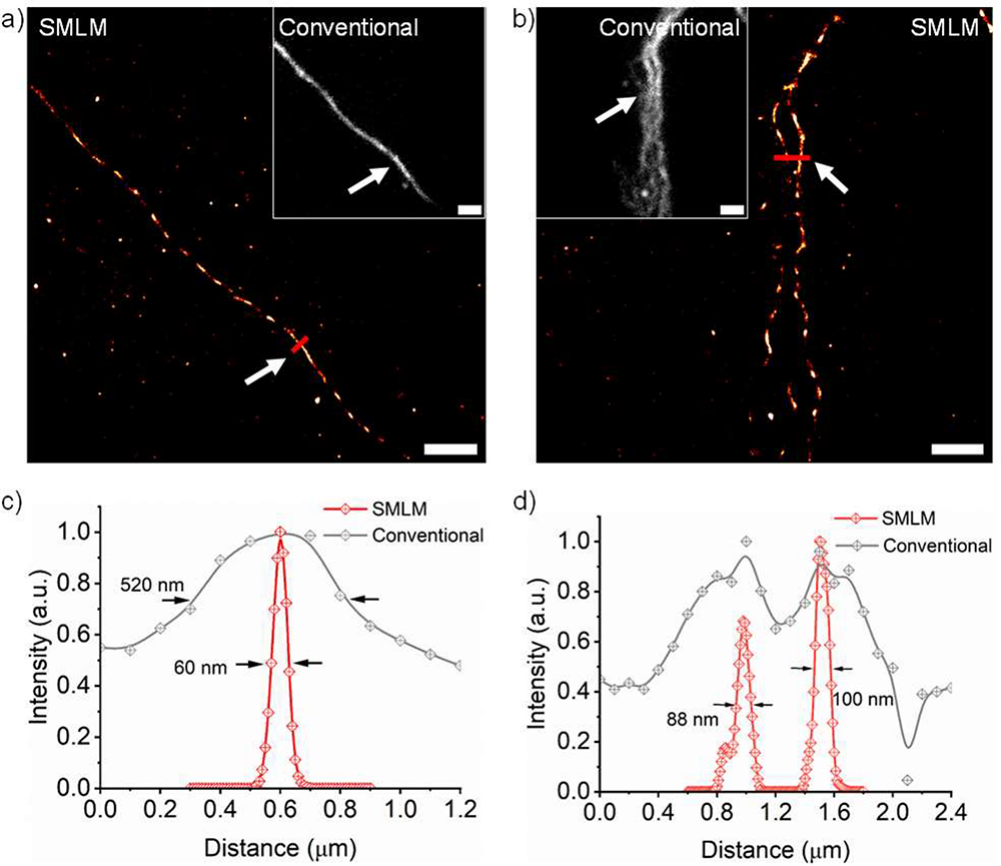
SMLM imaging of nascent DNA labeled by **PMIP-N**_**3**_ in PBS solution. (a, b) Reconstructed SMLM images of nascent DNA and corresponding conventional wide-field images (insets). (c) Profile of red line marked in a, white arrows indicating the position. (d) Profile of red line marked in b, white arrows indicating the position. Scale bars: 2 μm.

In summary, we have developed a bright NIR fluorophore, **PMIP**, with absorption and emission reaching into the NIR region. **PMIP** displays an excellent intrinsic photoblinking with low on–off duty cycles of ∼10^–3^ and high photon numbers of ∼1800. As a proof of concept of the NIR live-cell experiment, super-resolution SMLM imaging of lysosomes in living cells is demonstrated with **PMIP-OH** under physiological conditions. Moreover, metabolically labeled nascent DNA is site-specifically detected by **PMIP-N**_**3**_ via the click reaction, enabling SMLM imaging with a 9-fold resolution improvement compared to conventional wide-field imaging. These results illustrate the potential of **PMIP**-based NIR fluorophores to expand the biological applications of SMLM, particularly in live-cell and *in vivo* imaging.

## Supporting Information Available

Synthesis and characterizations of new compounds; experimental details and data analysis; supplementary figures and NMR spectra (PDF)
