# Supplementary material for: Near-Infrared Perylenecarboximide Fluorophores for Live-Cell Super-Resolution Imaging
Source: J Am Chem Soc. 2024 Mar 5;146(11):7135–9. doi: 10.1021/jacs.3c13368 (PMC10958508; doi:10.1021/jacs.3c13368)
Supplement: Supplementary file 1 — ja3c13368_si_001.pdf [file ja3c13368_si_001.pdf]

Supporting Information

Near-Infrared Perylenecarboximide Fluorophores for Live-Cell Super-Resolution Imaging

Ze-Hua Wu[†,§,‡], Xingfu Zhu,[†,‡] Qiqi Yang,[†] Yulian Zagranyarski,[†] Krishna Mishra,[§] Hilmar Strickfaden,[▽] Ronald P. Wong,[#] Thomas Basché,[§] Kaloian Koynov,[†] Mischa Bonn,[†] Chen Li,[†]* Xiaomin Liu,[†]* and Klaus Müllen[†,§]*

[†] Max Planck Institute for Polymer Research, Ackermannweg 10, 55128 Mainz, Germany

[§] Department of Chemistry, Johannes Gutenberg-University, 55099 Mainz, Germany

[▽] Katz Group Center, University of Alberta, Edmonton, AB, T6G 2T9, Canada

[#] Institute of Molecular Biology (IMB), Ackermannweg 4, 55128 Mainz, Germany

Table of Contents

1. General methods S1

2. Synthesis details S1

3. Coverslip cleaning S6

4. Preparation of PMIP sample for single-molecule blinking property measurement in air S6

5. Preparation of nanoscale crevices coated with PMIP molecules for SMLM imaging in air S6

6. Co-localization experiments of PMIP-OH with LysoTracker Green and MitoTracker Green S6

7. Live cell SMLM imaging of lysosomes with PMIP-OH S7

8. Labeling and imaging of newly synthesized DNA S7

9. Measurement of single-molecule blinking and super-resolution imaging S8

10. SMLM image data analysis S9

11. Supplementary table and figures S10

12. NMR spectra S16

13. References S22

1. General methods

Instrument: All reactions of air- or moisture-sensitive compounds were carried out under argon atmosphere using standard Schlenk line techniques. Nuclear Magnetic Resonance (NMR) spectra were taken in deuterated solvents using Bruker AVANCE III 300, Bruker AVANCE III 400, or Bruker AVANCE III 700 MHz NMR spectrometers. The ^{1}H and ^{13}C chemical shifts (δ) were recorded in parts per million and the TMS signal was used as an internal standard. Coupling constants (J) were measured in Hertz with multiplicities explained by the following abbreviations: s = singlet, d = doublet, t = triplet, dd = double of doublets, m = multiplet, br = broad. Melting points were determined on a Büchi hot stage apparatus. High-resolution mass spectra (HRMS) were recorded by atmospheric pressure chemical ionization (APCI) on a MicrOTOF-QII instrument and by matrix-assisted laser decomposition/ionization (MALDI) using 7,7,8,8-tetracyanoquinodimethane (TCNQ) as matrix on a Bruker Reflex II-TOF spectrometer. Absorption spectra were measured on a Perkin Elmer Lambda 900 spectrophotometer, fluorescence spectra on a Horiba Jobin Yvon FluoroMax-4 NIR spectrophotometer. All Chemical reagents and solvents were purchased from Aldrich, Acros, ABCR, TCI and used as received without further purification unless otherwise noted. Thin layer chromatography (TLC) was done on silica gel-coated aluminum sheets with an F254 indicator and column chromatography separation was performed with silica gel (particle size 0.063-0.200 mm). Fluorescence correlation spectroscopy (FCS) measurements were performed on a commercial setup (Carl Zeiss, Germany) consisting of the modules LSM510, ConfoCor 2 and an inverted microscope model Axiovert 200 equipped with a C-Apochromat 40×, NA 1.2 water immersion objective. A HeNe laser (633 nm) were used for excitation and the emission was collected after filtering with a LP650 long pass filter.

2. Synthesis details

8,9-Bis(benzylamino)-5,6,11,12-tetrachloro-2-(2,6-diisopropylphenyl)-1H-benzo[5,10]anthra[2,1,9-def]isoquinoline-1,3(2H)-dione (2a)

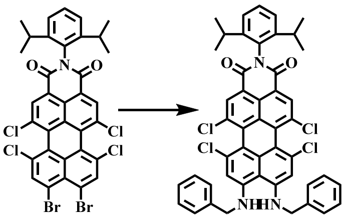

2a

To a suspension of 8,9-dibromo-5,6,11,12-tetrachloro-N-2,6-diisopropylphenyl perylene dicarboximide (0.28 mmol, 200 mg) in 5 ml methylpyrrolidone, benzylamine (1.12 mmol, 109 mg) and triethylamine (0.61 mmol, 62 mg) were added. The mixture was heated at 90°C for 0.5 h under argon atmosphere. After cooling, the reaction mixture was poured into hexane (30 ml), and the precipitate was filtered. The crude product was dissolved in a minimum amount of dichloromethane and added into hexane

(40 ml) to form a blue precipitate which was then collected through filtration. After oven drying at 100 °C, the title product was obtained as a blue solid (0.15 g; 70%). ^{1}H NMR (300 MHz, CD_2Cl_2, 293 K, ppm): δ 8.60 (s, 2H, Ar-H), 7.56 (t, 1H, J = 9.0 Hz, Ar-H), 7.40 (m, 8H, Ar-H), 7.32 (m, 4H, Ar-H), 7.04 (s, 2H, Ar-H), 6.20 (s, 2H, Ar-H), 4.46 (s, 4H, CH2), 2.83 (m, 2H, CH), 1.19 (dd, 12H, J = 1.0 Hz, CH3). ^{13}C NMR (Spin-echo,126 MHz, CD_2Cl_2, 293 K, ppm): δ 163.27, 149.13, 146.13, 138.00, 136.79, 135.85, 132.55, 132.07, 131.33, 129.98, 129.37, 129.08, 128.04, 127.93, 123.02, 119.62, 114.78, 110.98, 49.28, 29.06, 23.69. HRMS (MALDI+) *m/z*: calcd for C48H37Cl4N3O2 [M+]: 827.1640; Found: 827.1651.

1,3-Dibenzyl-5,6,12,13-tetrachloro-9-(2,6-diisopropylphenyl)-2,2-dimethyl-2,3-dihydropyrido[3',4',5':6,7]phenaleno[1,2,3-gh]perimidine-8,10(1H,9H)-dione (PMIP)

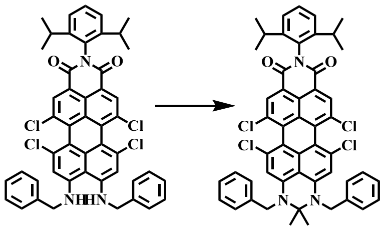

To the solution of compound **2a** (0.17 mmol, 140 mg) in dry acetone (5 mL), trifluoroacetic acid (0.1 mL) was added and refluxed for 12 h under argon atmosphere. The reaction mixture was cooled to room temperature and neutralized with triethylamine. The precipitate was filtered, washed with water, and dried. The crude product was purified by column chromatography using dichloromethane as an eluent on silica gel. After oven drying at 100 °C, the product was obtained as a blue solid (0.11 g; 75%). ^{1}H NMR (300 MHz, CD_2Cl_2, 293 K, ppm): δ 8.57 (s, 2H, N-H), 7.55 (t, 4H, J = 8.0 Hz, Ar-H), 7.46 (m, 8H, Ar-H), 7.38 (m, 4H, Ar-H), 6.82 (s, 2H, Ar-H), 4.99 (dd, 2H, J= 6.0 Hz, CH2), 2.81 (m, 2H, CH), 1.76 (s, 6H, CH3), 1.18 (dd, 12H, J= 6.0 Hz, CH3). ^{13}C NMR (400 MHz, CD_2Cl_2, 293 K, ppm): δ 163.30, 146.13, 145.11, 138.98, 137.33, 133.66, 132.63, 132.09, 131.43, 129.31, 129.13, 129.04, 127.56, 126.10, 124.61, 123.99, 118.53, 113.30, 109.26, 108.36, 76.29, 50.83, 29.04, 24.50, 23.70. HRMS (MALDI+) *m/z*: calcd for C51H41Cl4N3O2 [M+]: 867.1953; Found: 867.1936. Elem. Anal. calcd for C51H41Cl4N3O2: C, 70.43; H, 4.75; N, 4.83; Found: C, 70.27; H, 4.92; N, 5.05. Mp: 330.5-331.0 °C.

5,6,12,13-Tetrachloro-9-(2,6-diisopropylphenyl)-1,3-bis(2,4-dimethoxybenzyl)-2,2-dimethyl-2,3-dihydropyrido[3',4',5':6,7]phenaleno[1,2,3-gh]perimidine-8,10(1H,9H)-dione (3)

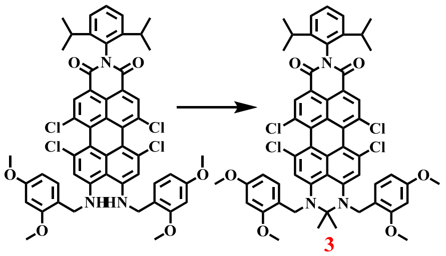

3

To the solution of compound **2b** (0.2 mmol, 190 mg) in dry acetone (5 mL), trifluoroacetic acid (0.2 mL) was added and refluxed for 12 h under argon atmosphere. The reaction mixture was cooled to room temperature and neutralized with triethylamine. The precipitate was filtered, washed with water, and dried. The crude product was purified by column chromatography using dichloromethane as an eluent on silica gel. After oven drying at 100 °C, the product was obtained as a blue solid (0.15 g; 80%). ^{1}H NMR (400 MHz, CD_2Cl_2, 293 K, ppm): δ 8.46 (s, 2H, Ar-H), 7.44 (t, 1H, J = 8.4 Hz, Ar-H), 7.28 (d, 2H, J= 8.4 Hz, Ar-H), 7.12 (d, 2H, J= 8.4 Hz, Ar-H), 6.45 (d, 2H, J= 3.2 Hz, Ar-H), 6.34 (dd, 2H, J= 8.4 Hz, Ar-H), 5.85 (s, 2H, CH), 4.20 (dd, 4H, J= 6.0 Hz, CH), 3.90 (s, 6H, CH3), 3.66 (s, 6H, CH3), 2.71 (m, 2H, CH), 1.44 (s, 6H, CH3), 1.08 (m, 12H, CH3). ^{13}C NMR (400 MHz, CD_2Cl_2, 293 K, ppm): δ 163.07, 159.81, 156.75, 145.82, 142.71, 138.83, 132.87, 132.38, 132.05, 131.67, 130.11, 129.23, 128.69, 124.87, 124.41, 118.70, 117.67, 112.99, 105.59, 98.49, 66.19, 55.86, 54.96, 29.69, 29.04, 28.63, 25.19, 23.72. HRMS (MALDI+) *m/z*: calcd for C55H49Cl4N3O6 [M+]: 987.2375; Found: 987.2368.

1,3-Dibenzyl-5,6,12,13-tetrachloro-9-(2,6-diisopropylphenyl)-2,2-dimethyl-2,3-dihydropyrido[3',4',5':6,7]phenaleno[1,2,3-gh]perimidine-8,10(1H,9H)-dione (PMIP-OH)

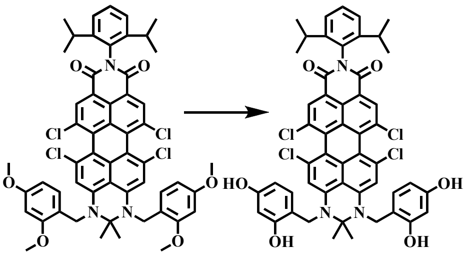

PMIP-OH

To the solution of **3** (0.17 mmol, 140 mg) in dry dichloromethane (10 mL), BBr_3 (0.1 mL) was added at 0°C under argon atmosphere. The mixture was stirred at room temperature for 24 h. Methanol (1 ml) was added to quench the reaction and the solvent was completely evaporated under reduced pressure. The crude product was purified by preparative GPC column with tetrahydrofuran as eluent. After oven drying at 100 °C, the title product was obtained as a blue solid (0.08 g; 60%). ^{1}H NMR (400 MHz, THF-d8, 293 K, ppm): δ 8.85 (s, 2H, Ar-H), 8.33 (s, 2H, Ar-H), 7.90 (s, 2H, Ar-H), 7.29 (t, 1H, J = 8.4 Hz, Ar-H), 7.18 (d, 2H, J = 8.4 Hz, Ar-H), 6.86 (d, 2H, J = 8.4 Hz, Ar-H), 6.73 (s, 2H, Ar-H), 6.15 (d, 2H, J= 2.4 Hz, Ar-H), 6.04 (dd, 2H, J= 8.4 Hz, Ar-H), 4.13 (dd, J= 6.8 Hz, 2H, CH), 2.72 (m, 2H, CH), 1.04 (dd, 12H, J= 6.8 Hz, CH3). ^{13}C NMR (400 MHz, THF-d8, 293 K, ppm): δ 161.78, 156.66, 153.33, 145.44, 143.23, 140.90,

132.88, 131.55, 129.72, 128.36, 127.90, 123.28, 112.14, 106.90, 101.50, 28.56, 27.25. HRMS (MALDI+) *m/z*: calcd for C51H41Cl4N3O6 [M+]: 931.1749; Found: 931.1735. Elem. Anal. calcd for C51H41Cl4N3O6: C, 65.60; H, 4.43; N, 4.50; Found: C, 65.50; H, 4.35; N, 4.36. Mp: 350.5-351.2°C.

1,3-Dibenzyl-5,6,12,13-tetrachloro-9-(4-methoxyphenyl)-2,2-dimethyl-2,3-dihydropyrido[3',4',5':6,7]phenaleno[1,2,3-gh]perimidine-8,10(1H,9H)-dione (4)

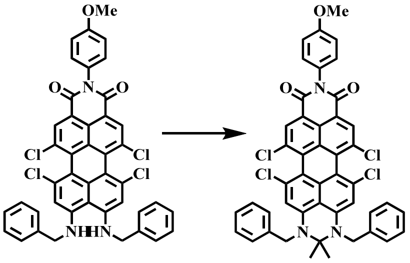

4

Following the procedure of **PMIP**, the crude product was purified by column chromatography using dichloromethane as an eluent on silica gel. After oven drying at 100 °C, the product was obtained as a blue solid (70%). ^{1}H NMR (400 MHz, CD_2Cl_2, 293 K, ppm): δ 8.54 (s, 2H, Ar-H), 7.48 (m, 8H, Ar-H), 7.38 (t, 2H, J= 8.4 Hz, Ar-H), 7.26 (d, 2H, J= 8.4 Hz, Ar-H), 7.12 (d, 2H, J= 8.4 Hz, Ar-H), 6.81 (s, 2H, Ar-H), 4.97 (dd, 4H, J= 2.8 Hz, CH), 3.92 (s, 3H, CH3), 1.76 (s, 6H, CH3). ^{13}C NMR (400 MHz, CD_2Cl_2, 293 K, ppm): δ 163.45, 159.58, 145.04, 138.90, 132.34, 131.98, 129.73, 129.13, 129.03, 128.34, 127.54, 126.09, 124.24, 118.84, 114.44, 113.32, 109.22, 108.36, 76.25, 55.50, 50.83, 24.48. HRMS (MALDI+) *m/z*: calcd for C46H31Cl4N3O3 [M+]: 813.1120; Found: 813.0880.

1,3-Dibenzyl-5,6,12,13-tetrachloro-9-(4-hydroxyphenyl)-2,2-dimethyl-2,3-dihydropyrido[3',4',5':6,7]phenaleno[1,2,3-gh]perimidine-8,10(1H,9H)-dione (5)

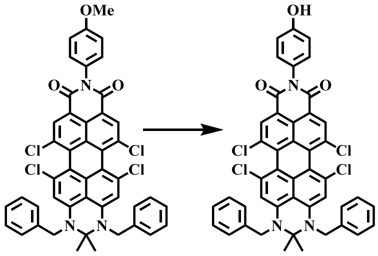

5

To the solution of **4** (0.2 mmol, 160 mg) in dry dichloromethane (20 mL), BBr_3 (0.2 mL) was added at 0°C under argon atmosphere. The mixture was then stirred at room temperature for 24 h. Methanol (1 ml) was added to quench the reaction and the solvent was completely evaporated under reduced pressure. The crude product was purified by column chromatography using ethyl acetate and hexane (1:1) as eluent on silica gel. After oven drying at 100 °C, the product was obtained as a blue solid (0,13 g; 85%). ^{1}H NMR (400 MHz, THF-d8, 293 K, ppm): δ 10.72 (s, 2H, OH), 8.30 (s, 2H, Ar-H), 7.35 (d, 4H, J = 8.0 Hz, Ar-H), 7.27 (t, 6H, J = 8.0 Hz, Ar-H), 7.16 (t, 2H, J = 8.0 Hz, Ar-

H), 6.96 (d, 2H, J = 8.0 Hz, Ar-H), 6.74 (s, 2H, Ar-H), 6.73 (d, 2H, J= 8.0 Hz, Ar-H), 4.96 (dd, J= 2.8 Hz, 4H, CH2), 1.18 (s, 6H, CH3). HRMS (MALDI+) *m/z*: calcd for C45H29Cl4N3O3 [M+]: 799.0963; Found: 799.0456.

1,3-Dibenzyl-9-(4-(3-bromopropoxy)phenyl)-5,6,12,13-tetrachloro-2,2-dimethyl-2,3-dihydropyrido[3',4',5':6,7]phenaleno[1,2,3-gh]perimidine-8,10(1H,9H)-dione (6)

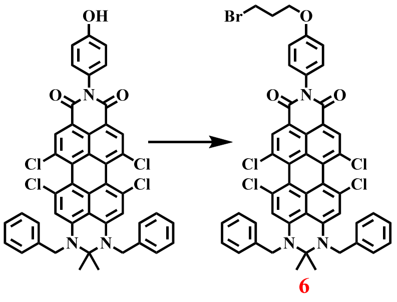

A mixture of **5** (0.1 mmol, 80 mg), 1,3-dibromopropane (0.2 mmol, 40 mg) and potassium carbonate (0.2 mmol, 27 mg) in 10 ml dry DMF was stirred at room temperature for 24 h under argon atmosphere. The reaction mixture was poured into water (50 ml) and the precipitate was filtered. The crude product was purified by column chromatography using DCM and hexane (1:1) as eluent on silica gel. After oven drying at 100 °C, the product was obtained as a blue solid (0.06 g; 70%). ^{1}H NMR (400 MHz, CD_2Cl_2, 293 K, ppm): δ 8.54 (s, 2H, Ar-H), 7.46 (m, 8H, Ar-H), 7.38 (t, 2H, J= 8.0 Hz, Ar-H), 7.26 (d, J= 8.0 Hz, Ar-H), 7.13 (d, J= 8.0 Hz, Ar-H), 6.81 (s, 2H, Ar-H), 4.98 (dd, J= 2.8 Hz, 4H, CH2), 4.25 (t, J= 6.0 Hz, 2H, CH2), 3.72 (t, J= 6.0 Hz, 2H, CH2), 2.44 (m, J= 6.0 Hz, 2H, CH2), 1.76 (s, 6H, CH3). ^{13}C NMR (101 MHz, CD_2Cl_2): δ 163.44, 158.70, 145.05, 138.91, 137.36, 135.82, 133.62, 132.34, 131.99, 129.79, 129.13, 129.03, 127.54, 126.09, 125.40, 118.82, 115.00, 113.32, 109.22, 108.36, 76.25, 65.70, 34.09, 32.40, 31.93, 30.19, 30.07, 29.69, 24.48, 22.69, 20.82, 13.88. HRMS (MALDI+) m/z: calcd for C48H34BrCl4N3O3 [M+]: 919.0538; Found: 919.0497.

3-Azido-N-(3-(4-(1,3-dibenzyl-5,6,12,13-tetrachloro-2,2-dimethyl-8,10-dioxo-1,3,8,10-tetrahydropyrido[3',4',5':6,7]phenaleno[1,2,3-gh]perimidin-9(2H)-yl)phenoxy)propyl)-N,N-dimethylpropan-1-ammonium bromide (PMIP-N$_3$)

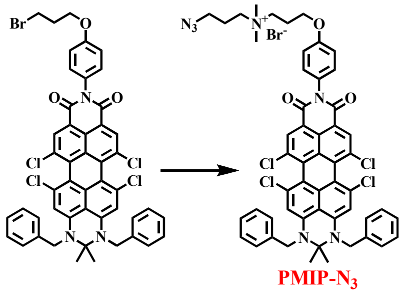

A mixture of **6** (0.01 mmol, 9 mg) and 3-azido-N,N-dimethyl-1-propanamine (0.5 mmol, 60 mg) in 3 ml dry acetonitrile was stirred at 60°C for 3 h under argon atmosphere. After cooling down, the solution was concentrated using airflow, and the

precipitate was subsequently filtered. After oven drying at 50 °C, the product was obtained as a blue solid (6.5 mg; 70%). ^{1}H NMR (400 MHz, CD_2Cl_2, 293 K, ppm): δ 8.40 (s, 2H, Ar-H), 7.33 (m, 8H, Ar-H), 7.25 (t, 2H, J= 8.0 Hz, Ar-H), 7.15 (d, 2H, J= 8.0 Hz, Ar-H), 7.04 (d, J= 8.0 Hz, Ar-H), 6.68 (s, 2H, Ar-H), 4.84 (dd, J= 2.8 Hz, 4H, CH2), 3.48 (dd, J= 5.2 Hz, 4H, CH2), 3.28 (s, 6H, CH3), 2.98 (m, 4H, CH2), 2.07 (m, 4H, CH2), 1.63 (s, 6H, CH3). ^{13}C NMR (101 MHz, CD_3OD): δ 163.58, 158.46, 146.02, 139.14, 137.62, 132.52, 132.06, 131.89, 131.60, 129.67, 128.69, 128.60, 127.11, 125.85, 119.44, 115.63, 112.64, 109.26, 107.95, 75.42, 54.92, 49.66, 41.52, 23.85, 23.21, 22.76, 22.56. HRMS (MALDI+) m/z: calcd for C53H46Cl4N7O3 [M-Br]: 968.2416; Found: 968.2399.

3. Coverslip cleaning

Coverslip cleaning was conducted according to a previous literature method.[1,2] In brief, the coverslips were sonicated in 1% Micro 90 alkaline cleaning solution for 15 min. The coverslips were then rinsed three times with Milli-Q water and finally dried in a nitrogen flow. Finally, those coverslips were cleaned by an oxygen-plasma cleaner (250 W, 10 min). The gridded coverslip (IBIDI, Cat. No: 10817) underwent the same cleaning process. These coverslips contained nanoscale crevices during preparation.

4. Preparation of PMIP, PMIP-OH and PMIP-N_3 samples for single-molecule blinking property measurement in air

The DMSO solutions of **PMIP**, **PMIP-OH** and **PMIP-N_3** (10^{-11} M, 10 μL) were spin-coated on the cleaned coverslips, respectively. In brief, the coverslips were first spun at 2,000 rpm for 20 s and then at 4,000 rpm for 40 s. The samples were dried on a hot plate by heating at 70 °C for 15 min.

5. Preparation of nanoscale crevices coated with PMIP molecules for SMLM imaging in air

The toluene solution of **PMIP** (10^{-7} M, 10 μL) was drop-casted on the cleaned gridded coverslip. Subsequently, the coverslip was dried on a hot plate by heating at 70 °C for 15 min.

6. Co-localization experiments of PMIP-OH with LysoTracker Green and MitoTracker Green

Lysosomes: U2OS cells were cultured in Dulbecco's Modified Eagle's Medium (DMEM) supplemented with 10% fetal bovine serum (FBS), in a 5% CO_2 humidified incubator at 37 °C. The cells were then plated into 35 mm diameter glass bottom Petri dishes (IBIDI) and incubated overnight under the same conditions. Live U2OS cells were stained with 1 μM **PMIP-OH** in DMEM (supplement 10% FBS) medium for one, three, twenty-four hours, respectively, and then rinsed three times in phenol red-free

DMEM (each time for five minutes). Subsequently, 75 nM LysoTracker Green in DMEM was added. After 30 minutes, the cells were washed twice with DMEM. Finally, the washing medium was replaced with DMEM (supplement 10% FBS) before imaging. **PMIP-OH** was excited by a 635 nm laser and LysoTracker Green was excited by a 475 nm laser.

Mitochondria: U2OS cells were cultured in DMEM supplemented with 10% FBS, in a 5% CO_2 humidified incubator at 37 °C. The cells were then plated into 35 mm diameter glass bottom Petri dishes and incubated overnight under the same conditions. Live U2OS cells were stained with 1 μM **PMIP-OH** in DMEM (supplement 10% FBS) medium for twenty-four hours, and then rinsed three times in phenol red-free DMEM (each time for five minutes). Subsequently, 75 nM MitoTracker Green in DMEM was added. After 30 minutes, the cells were washed twice with DMEM. Finally, the washing medium was replaced with DMEM (supplement 10% FBS) before imaging. **PMIP-OH** was excited by a 635 nm laser and MitoTracker Green was excited by a 475 nm laser.

7. Live cell SMLM imaging of lysosomes with PMIP-OH

U2OS cells were cultured in Dulbecco's Modified Eagle's Medium (DMEM) supplemented with 10% fetal bovine serum (FBS), in a 5% CO_2 humidified incubator at 37 °C. The cells were then plated into 35 mm diameter glass bottom Petri dishes (IBIDI) and incubated overnight under the same conditions. Live U2OS cells were stained with 1 μM **PMIP-OH** in DMEM (supplement 10% FBS) medium for three hours, and then rinsed three times in phenol red-free DMEM (each time for five minutes). Subsequently, 75 nM LysoTracker Green in DMEM was added. After 30 minutes, the cells were washed twice with DMEM. Finally, the washing medium was replaced with DMEM (supplement 10% FBS) before imaging. The conventional wide-field fluorescence images were first acquired. Afterward, 6,500 frames of living-cell imaging were recorded with a 642 nm laser excitation power of 1 kW/cm^2, at an exposure time of 23 ms.

8. Labeling and imaging of newly synthesized DNA

U2OS cells were cultured in Dulbecco's Modified Eagle's Medium (DMEM) supplemented with 10% fetal bovine serum (FBS), in a 5% CO_2 humidified incubator at 37 °C. The cells were plated into 35 mm diameter glass bottom Petri dishes (IBIDI) and incubated overnight under the same conditions. (2'S)-2'-Deoxy-2'-fluoro-5-ethynyluridine (F-ara-EdU) was then added (10 uM) for incubation for 24 h. Afterwards, the cells were washed with cold Dulbecco's phosphate-buffered saline (DPBS) three times, followed by detachment using trypsin. The detached cells were collected in an Eppendorf tube and centrifuged (500 r/min). The supernatant was discarded and the cells were resuspended in cold DPBS (0.2 mL). The Ultra Frost glass slide (positively charged surface) was cleaned with MilliQ water. Then 7.5 μl of lysis buffer (200 mM Tris-HCl pH7.4, 50 mM EDTA, 0.5% SDS) was gently mixed with 4 μl of cells directly

on the slide. Leave the slide horizontally for exactly 8.5 min at room temperature without moving.

The slide was manually tilted at an angle of 15°-30° to allow the liquid to slowly flow down the slide (taking approximately 5-10 minutes to reach the bottom). After drying completely in air, the slide was fixed with MeOH/acetic acid (volume ratio 3/1) for 2 hours at room temperature. The slide was then incubated for 2 hours at room temperature (protected from light) with the modified click mix solution: click-iT reaction buffer/DMSO/THF (volume ratio 4/3/3), additives, 2 uM **PMIP-N$_3$**, 0.5 mM $CuSO_4$ (click it reaction buffer, additives and $CuSO_4$ were from Click-iT EdU kit). The slide was washed three times with the click wash buffer (0.5 mM EDTA, 1% Tween 20, PBS) for 10 min, and washed with PBS twice. Co-staining with DAPI in PBS was carried out for 20 minutes, followed by two times washing with PBS. Subsequently, the coverslips were mounted in PBS and sealed with nail polish.

Control experiment: U2OS cells were cultured in Dulbecco's Modified Eagle's Medium (DMEM) supplemented with 10% fetal bovine serum (FBS), in a 5% CO_2 humidified incubator at 37 °C. The cells were plated into 35 mm diameter glass bottom Petri dishes (IBIDI) and incubated overnight under the same conditions. The cells were washed with cold DPBS three times, followed by detachment using trypsin. The detached cells were collected in an Eppendorf tube and centrifuged. The supernatant was discarded and the cells were resuspended in cold PBS (0.2 mL). The Ultra Frost glass slide (positive charged surface) was cleaned with MilliQ water. Then 7.5 µl of lysis buffer (200 mM Tris-HCl pH7.4, 50 mM EDTA, 0.5% SDS) was gently mixed with 4 µl of cells directly on the slide. Leave the slide horizontally for exactly 8.5 min at room temperature without moving. The slide was manually tilted at an angle of 15°-30° to allow the liquid to slowly flow down the slide (taking approximately 5-10 minutes to reach the bottom). After drying in air, the slide was fixed with MeOH/acetic acid (volume ratio 3/1) for 2 hours at room temperature. The slide then underwent the same click reaction with **PMIP-N$_3$** as previously described**.**

9. Measurement of single-molecule blinking and super-resolution imaging

Both single-molecule blinking measurements and super-resolution imaging were performed using the SR GSD microscope (Leica). 642 nm (500 mW) laser was selected for excitation. For the 642 nm laser, the excitation filter (637–647 nm/400–410 nm), the dichroic beam splitter (637–647 nm/400–410 nm), and the emission filter (660–760 nm/449–451 nm) were used. The objective lens HCX PL APO 160×1.43 NA Oil CORR-TIRF was selected for single-molecule measurements and super-resolution imaging. The microscope was equipped with an EMCCD camera (iXonDU-897, Andor). The camera settings were 10 MHz at 14 bit and a pre-amplification of 5.1. Please note here that the double bandwidth of the filters/beam splitter was chosen for

405 nm back pumping, such back pumping was used for SMLM imaging of nascent DNA. For single-molecule blinking measurements, 20,000 frames were recorded with an exposure time of 30 ms, EM gain of 100, 642 nm laser power of 5 kW/cm^2. For live-cell super-resolution imaging, 6,500 frames were taken with an exposure time of 23 ms, EM gain of 100, 642 nm laser power of 1 kW/cm^2. For super-resolution imaging of newly synthesized DNA labeled with **PMIP-N$_3$** in PBS, 20,000 frames were recorded with an exposure time of 30 ms, EM gain of 100, 642 nm laser power of 5 kW/cm^2.

10. SMLM image data analysis

All SMLM movies were analyzed with ThunderSTORM Plugin in ImageJ.[3] The peak intensity threshold was set as 1.7. Sigma > 90 and sigma < 150 were used to collect the true signal-molecule signal with a wavelength of 660 to 760 nm. Drift correction was conducted with cross-correlation function (number of bins of 3) in ThunderSTORM Plugin in ImageJ. All super-resolution images were reconstructed in ThunderSTORM. The pixel size of SMLM images of lysosomes and nascent DNA is 5 nm.

The photons per blinking event was calculated by localizing **PMIP** in each frame of the recorded imaging data. The localization was filtered based on the expected width of the signals and subsequently merged when they appeared in consecutive frames. To account for low photon yields that might lead to missed detections, the merging of one dark frame between two detections was allowed. As a spatial constraint, we applied a maximum distance of 80 nm, a large radius to ensure a proper merging of localizations with low photon counts. The resulting histogram of photon counts was subjected to fitting using a monoexponential function. The reported mean values were derived from the fit. The duty cycle, representing the fraction of time which a molecule resides in its fluorescent (on) state, was calculated according to the reference.[4]

The single-molecule fluorescence time trace was extracted by generating a maximum intensity projection of the recorded frames. Fluorescence signals in the projection were localized using the Thunderstorm-plugin in Fiji.[3,5] We then calculated the intensity trace for each localization throughout all frames of the raw data as the total background corrected intensity in a 7×7 region of interest (ROI) around the location coordinates. The local background for each localization in each frame was calculated within a 17×17 ROI. Pixel values bigger than 5 times the standard deviation within this ROI were considered fluorescence signals and excluded from the background calculation.

11. Supplementary table and figures

Table S1. Spectroscopic and photophysical data of **PMIP** and its derivatives

	ε[a] $M^{-1}cm^{-1}$	λ_{abs} [b] nm	λ_{em} [b] nm	$\Delta\lambda_{abs-em}$ nm	Φ_{fl}[c] %	Brightness ($\varepsilon\times\Phi_{fl}$) $M^{-1}cm^{-1}$
**PMIP**	6×10^4	660	732	72	60	3.6×10^4
**PMIP-N$_3$**	5.1×10^4	660	730	70	58	3.0×10^4
**PMIP-OH**	4.5×10^4	700	750	50	32	1.4×10^4

[a] Measured at λ_{abs}. [b] Measured in DMSO. [c] Measured in DMSO. Fluorescence reference: Rhodamine 800 in ethanol (Φ_{fl} = 0.25).

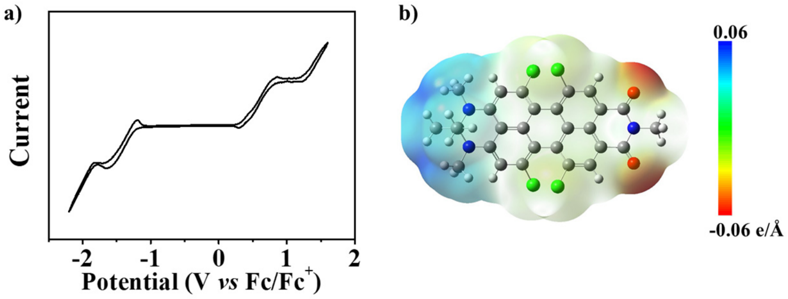

Figure S1. a) Cyclic voltammetry (CV) curve of **PMIP** in DCM with 0.1 M Bu_4NBF_4 as electrolyte at a scan rate of 100 mVs^{-1}. The measurement was carried out at room temperature with a conventional three-electrode configuration consisting of a platinum working electrode, a platinum wire auxiliary electrode, and a nonaqueous $Ag/AgNO_3$ reference electrode. b) The electrostatic potential (ESP) calculation of **PMIP** based on density functional theory (DFT) at the B3LYP/6-31G** level. As illustrated in Figure S1a, the onset oxidation (E^{ox}_{oneset}) and reduction potential (E^{red}_{oneset}) of **PMIP** are 0.3 and -1.1 V with reference to ferrocene/ferrocenium (Fc/Fc^+), respectively. The HOMO and LUMO energy levels (E_{HOMO}, E_{LUMO}) and the energy gap (E_{gap}) are accordingly calculated as -5.1, -3.6 eV and 1.5 eV, respectively. In comparison with the parental perylene dicarboximide (PDI) with E_{HOMO} of -6.1 eV, E_{LUMO} of -3.8 eV and E_{gap} of 2.3 eV, **PMIP** exhibits an increased E_{HOMO} and a narrowed E_{gap}, indicating pronounced intramolecular charge transfer (ICT) effect in **PMIP**. The ESP calculation of **PMIP** (Figure S1b) reveals the negative ESP regions on the carboximide moiety and the positive ESP regions on the pyrimidine moiety, suggesting the charge transfer characteristics and confirming the ICT.

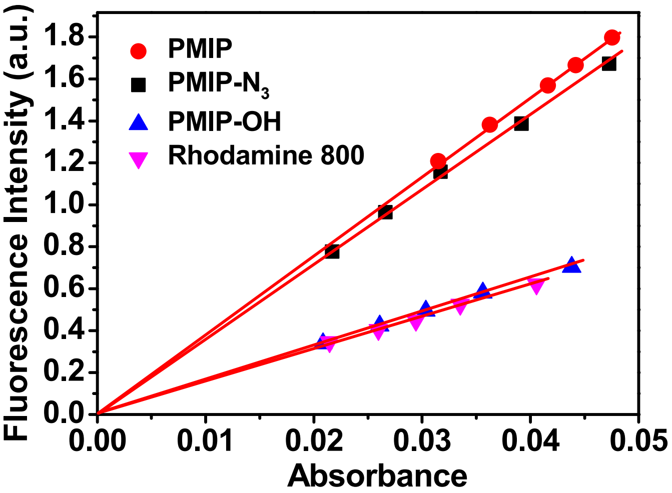

Figure S2. Integrated fluorescence intensity (for **PMIP**, **PMIP-N$_3$** and **PMIP-OH** in the wavelength range of 700-1000 nm; for Rhodamine 800, in the wavelength range of 650-1000 nm) versus absorbance (at 640 nm) of **PMIP**, **PMIP-N$_3$** and **PMIP-OH** in DMSO and Rhodamine 800 in ethanol.

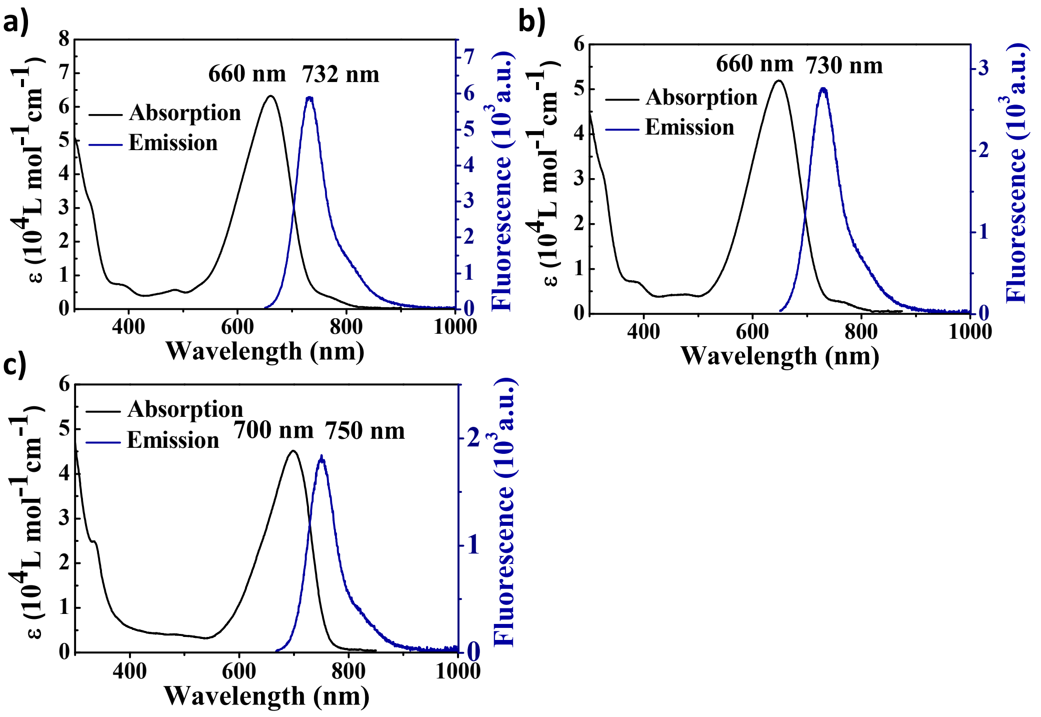

Figure S3. Absorption and emission spectra of a) **PMIP**, b**) PMIP-N$_3$**, and c**) PMIP-OH** in DMSO.

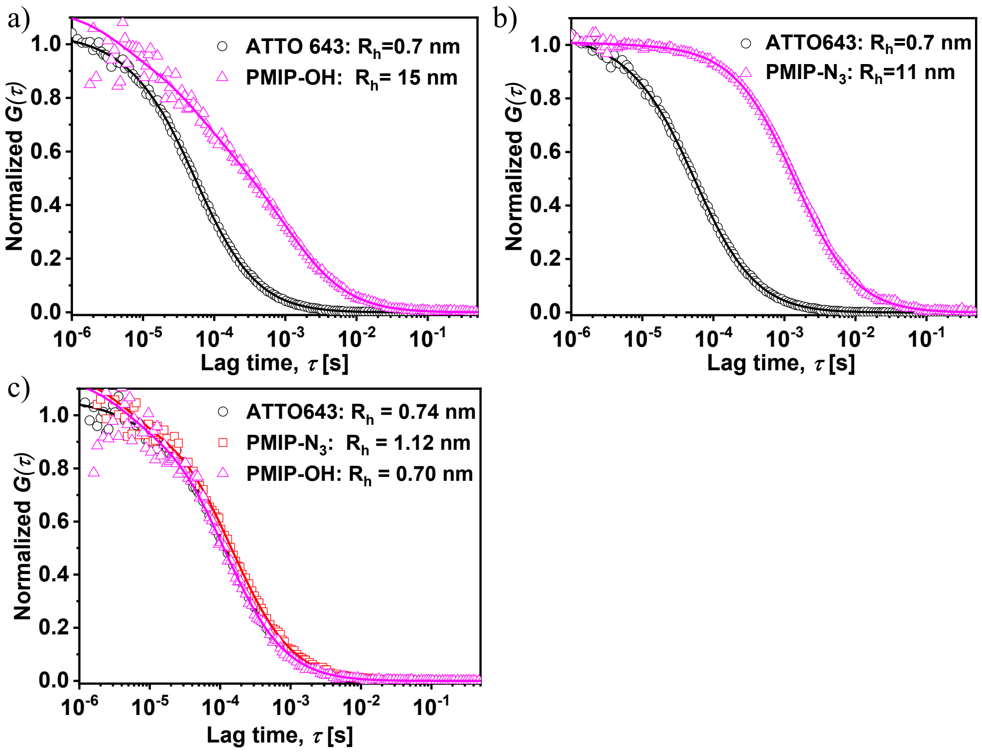

Figure S4. Characterization of the water solubility of **PMIP-N_3** and **PMIP-OH** by fluorescence correlation spectroscopy (FCS). a) Normalized FCS autocorrelation curves for **PMIP-OH** (magenta triangles) and a reference dye ATTO643 (black circles) measured in water. b) Normalized FCS autocorrelation curves for **PMIP-N_3** (magenta triangles) and a reference dye ATTO643 (black circles) measured in water. c) Normalized FCS autocorrelation curves for **PMIP-N_3** (red rectangles), **PMIP-OH** (magenta triangles) and a reference dye ATTO643 (black circles) measured in DMSO. In DMSO, the hydrodynamic radius (R_h) of all compounds is approximately 1 nm, indicating excellent solubility. In water, species with R_h of 15 and 11 nm were observed for **PMIP-OH** and **PMIP-N_3**, respectively, suggesting the formation of small aggregates.

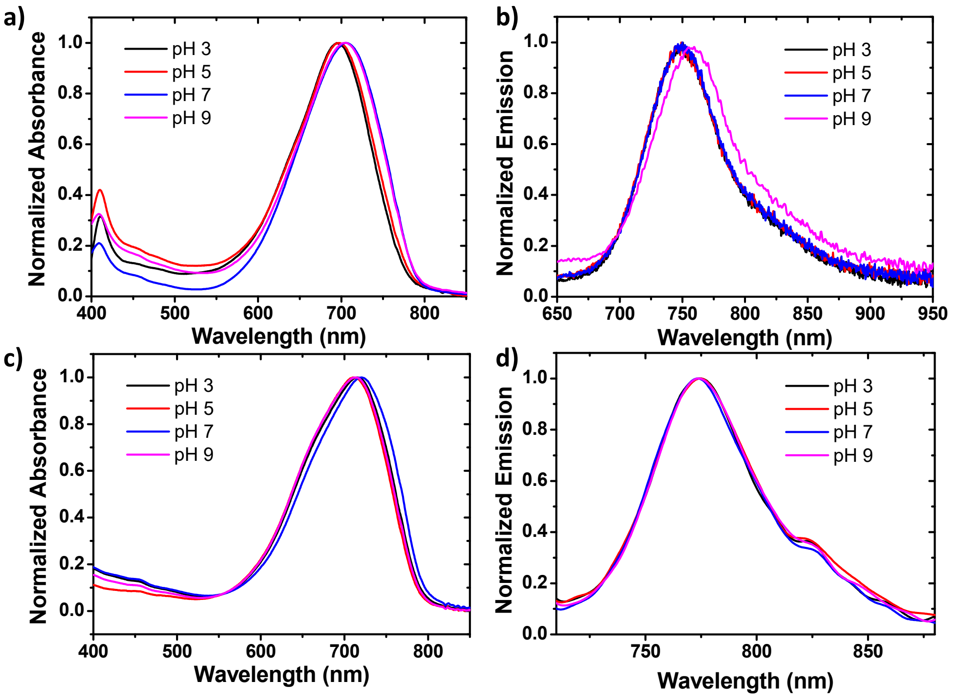

Figure S5. Absorption spectra of **PMIP-OH** in a) Dulbecco's modified Eagle's medium (DMEM) and c) phosphate-buffered saline (PBS) at different pH values. Emission spectra of **PMIP-OH** in b) DMEM and d) PBS at different pH values.

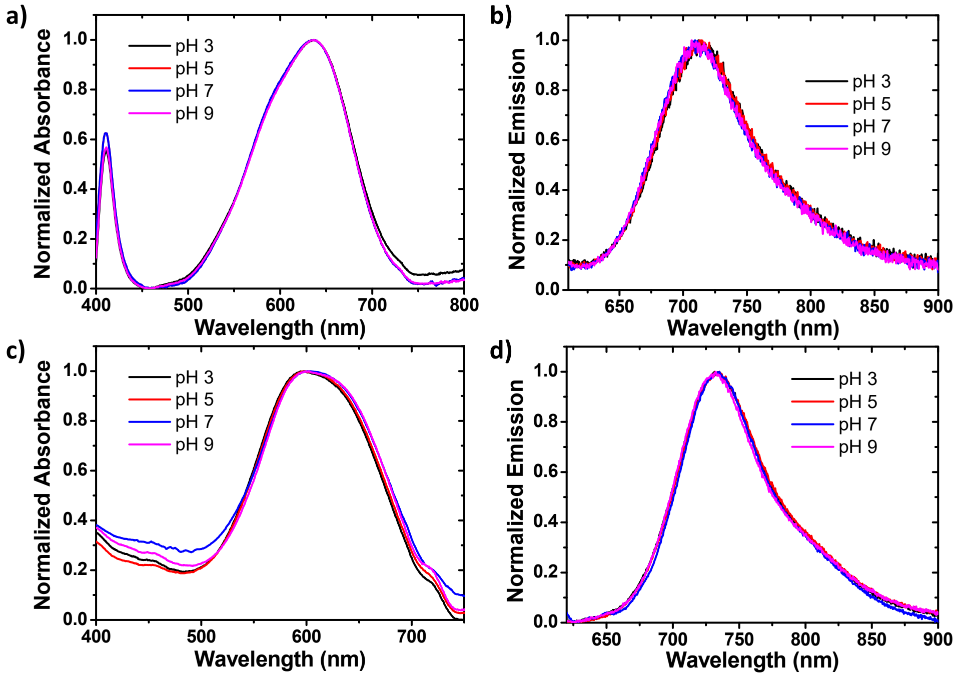

Figure S6. Absorption spectra of **PMIP-N$_3$** in a) DMEM and c) PBS at different pH values. Emission spectra of **PMIP-N$_3$** in b) DMEM and d) PBS at different pH values.

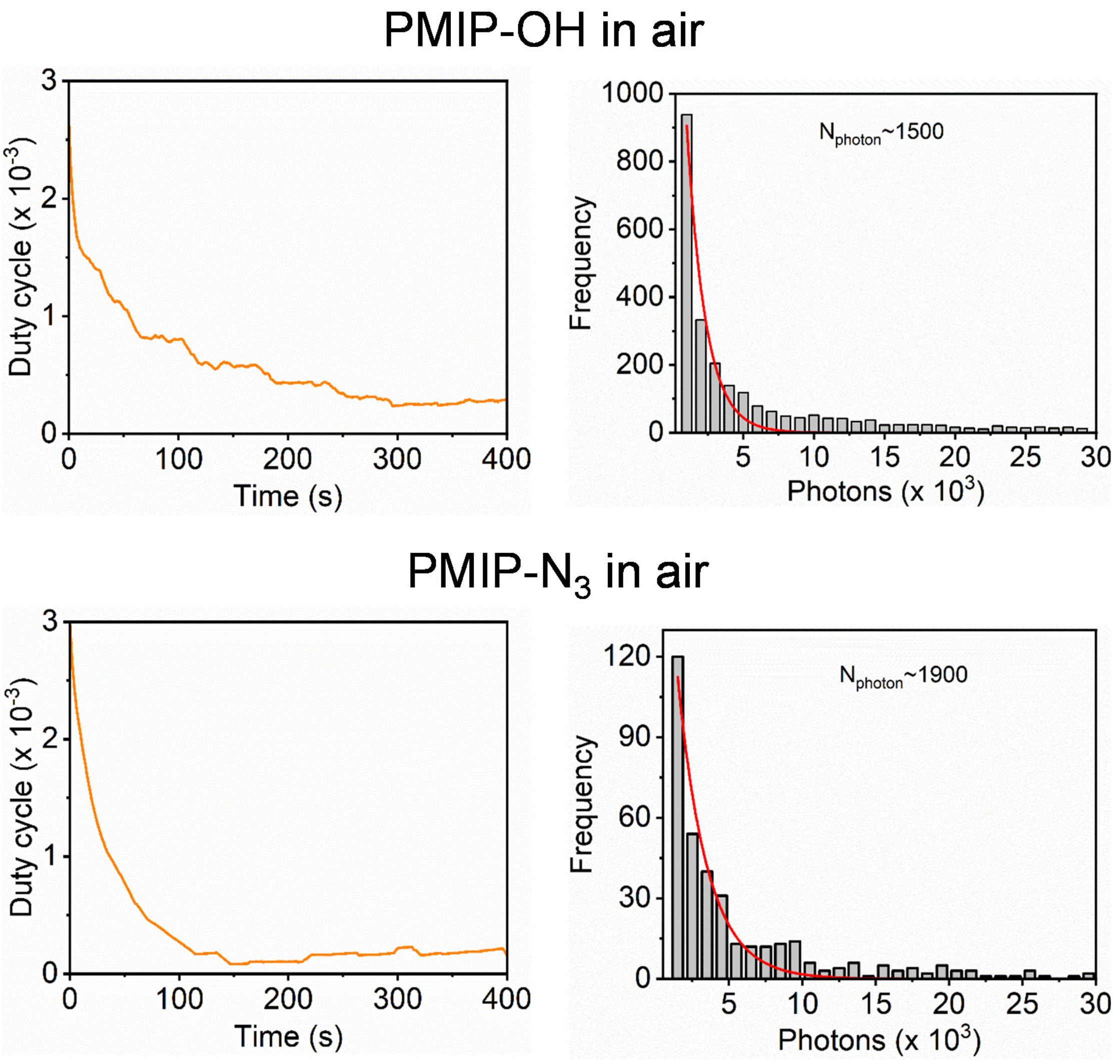

Figure S7. Blinking parameters of **PMIP-OH** and **PMIP-N_3** in air. Left: On-off duty cycle (fraction of time a molecule resides in its fluorescent state). Right: Histogram of detected photons per switching event and single-exponential fit.

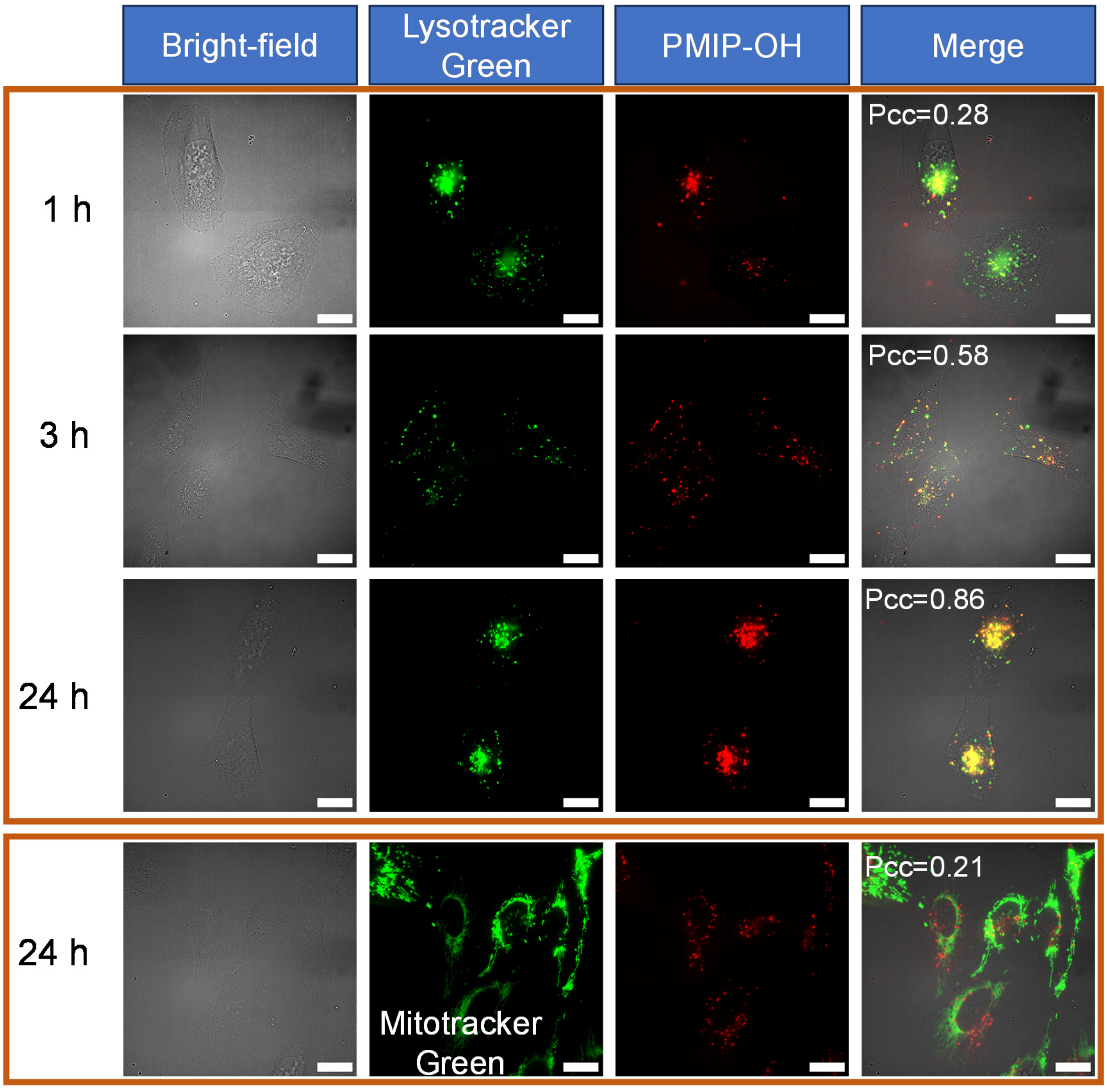

Figure S8. Co-localization of **PMIP-OH** with LysoTracker Green and MitoTracker Green in living U2OS cells. Top group images: the cells were incubated with **PMIP-OH** (1 μM) for 1, 3 and 24 hours, respectively and then co-stained with LysoTracker Green (75 nM) for 30 min. **PMIP-OH** was excited by a 635 nm laser, and LysoTracker Green was excited by a 475 nm laser. Bottom group images: The cells were incubated with **PMIP-OH** (1 μM) for 24 hours, and then co-stained with MitoTracker Green (75 nM) for 30 min. **PMIP-OH** was excited by a 635 nm laser, and MitoTracker Green was excited by a 475 nm laser. The Pearson's correlation coefficient (Pcc) of **PMIP-OH** with LysoTracker Green and MitoTracker Green are shown in the right panel images (Merge), using Coloc2 Plugin in Fiji (ImageJ). Taken together, **PMIP-OH** molecules can be accumulated in lysosomes through cellular uptake. Scale bars: 20 μm.

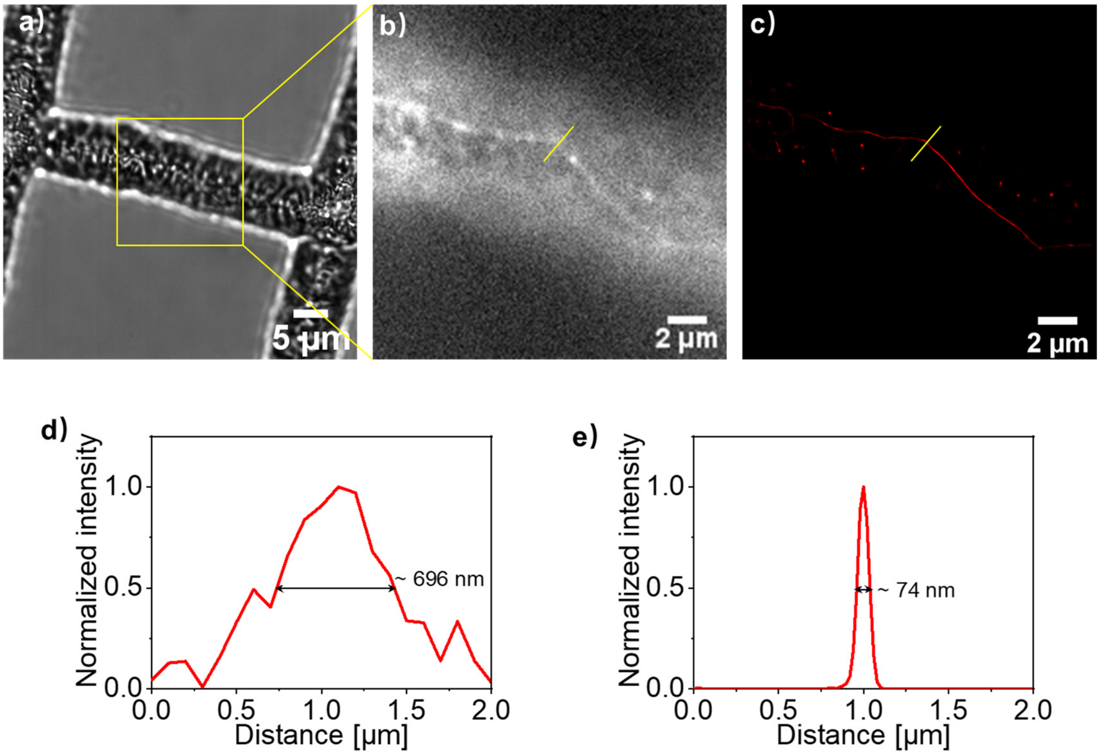

Figure S9. a) Bright-field image of gridded coverslip. b) Conventional wide-field image with **PMIP-OH** (yellow rectangle marked in a). c) Coverslip grid SMLM imaging with **PMIP-OH**. d) Profile of yellow line marked in b. e) Profile of yellow line marked in c. The gridded coverslip contained nanoscale crevices during preparing, making it suitable for nanoscale SMLM imaging as a proof of concept.

12. NMR spectra

^{1}H NMR (300MHz, CD2Cl2, 293 K, ppm) of **2a**

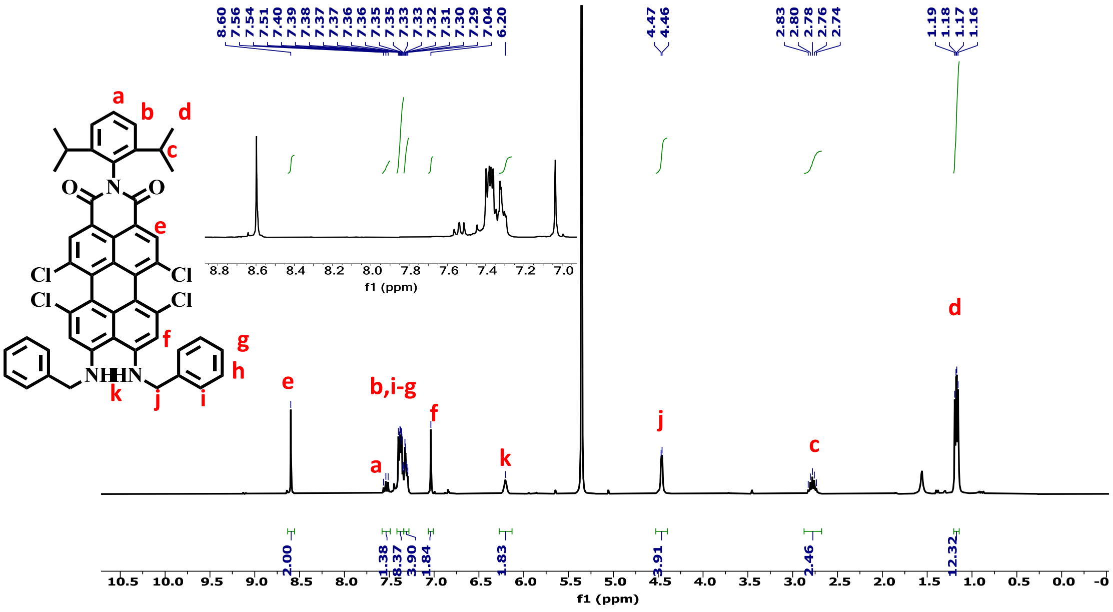

^{13}C NMR (Spin-echo,126 MHz, CD_2Cl_2, 293 K, ppm) of **2a**

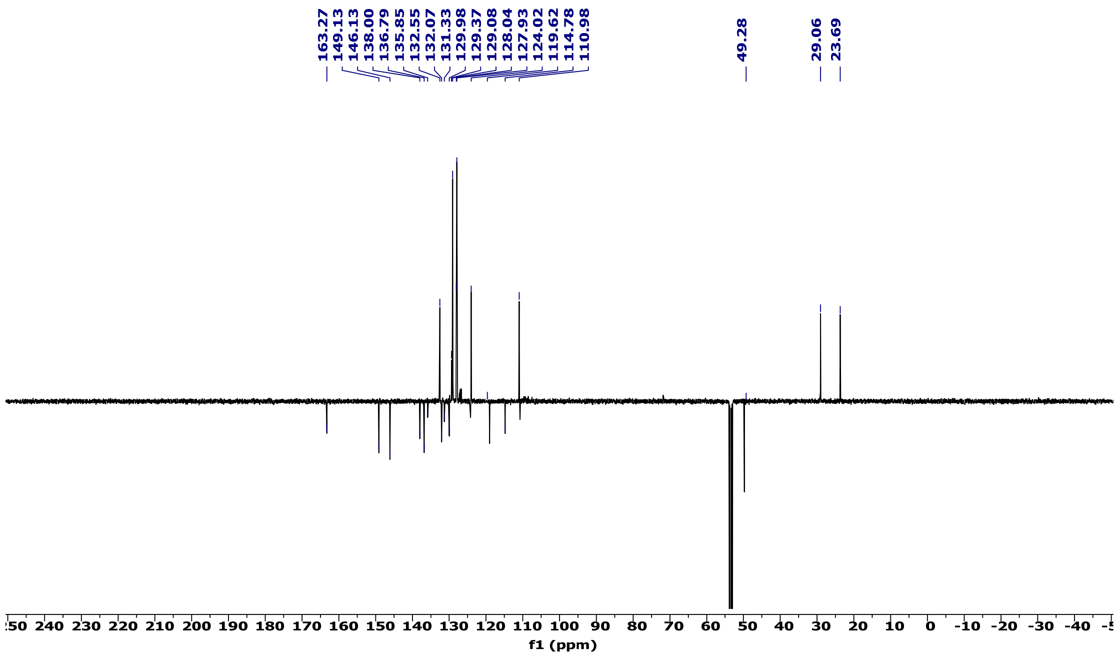

^{1}H NMR (300 MHz, CD_2Cl_2, 293 K, ppm) of **PMIP**

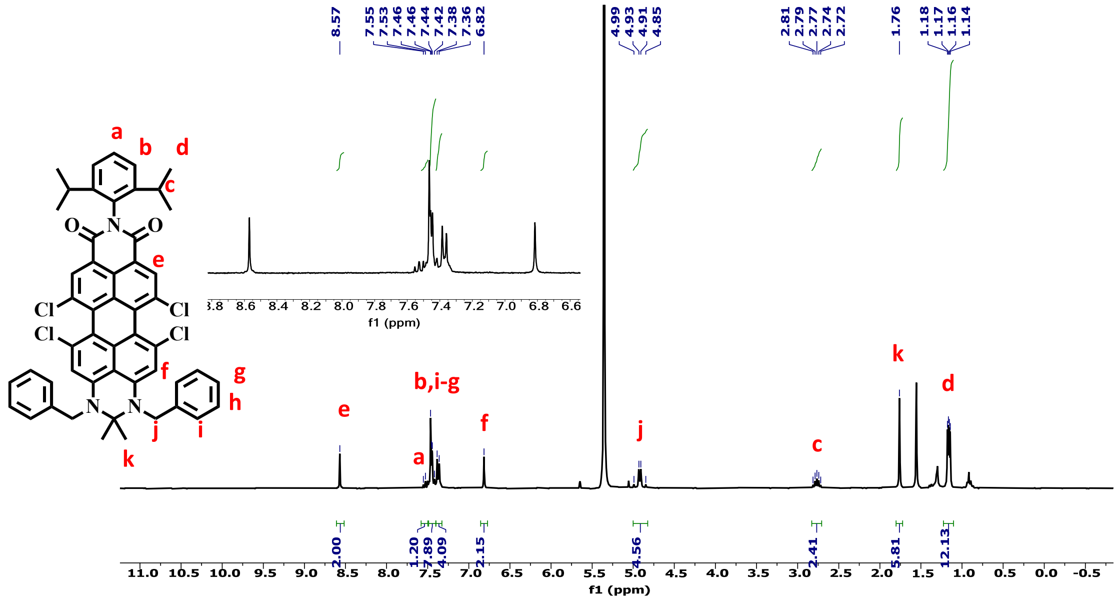

^{13}C NMR (400 MHz, CD_2Cl_2, 293 K, ppm) of **PMIP**

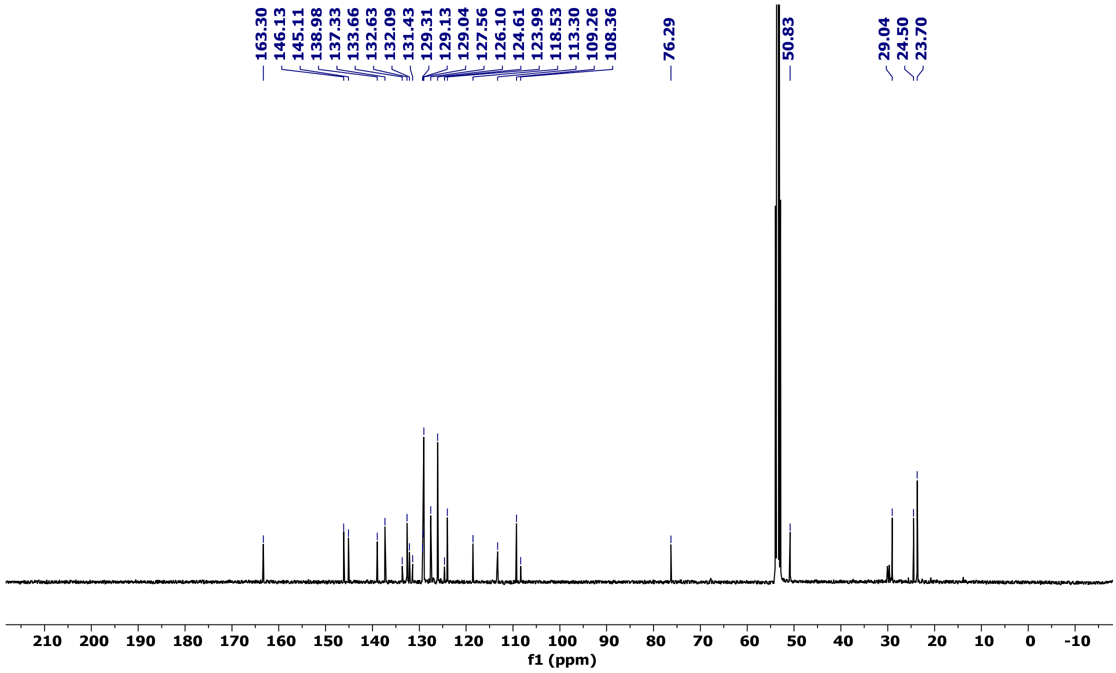

^{1}H NMR (400 MHz, CD_2Cl_2, 293 K, ppm) of **3**

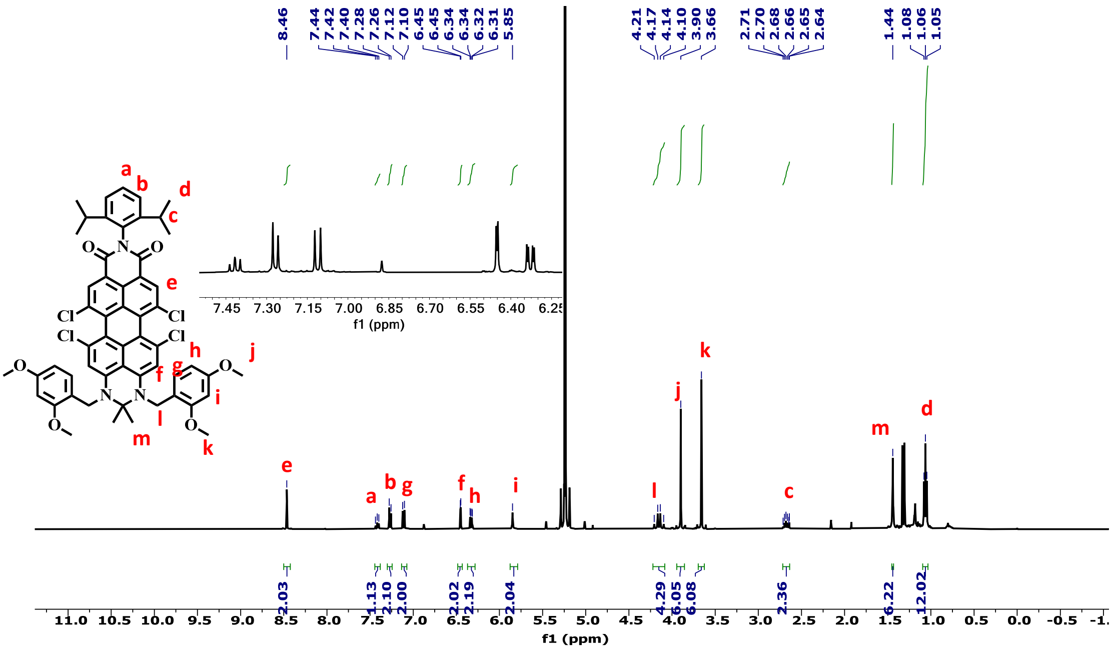

^{13}C NMR (400 MHz, CD_2Cl_2, 293 K, ppm) of **3**

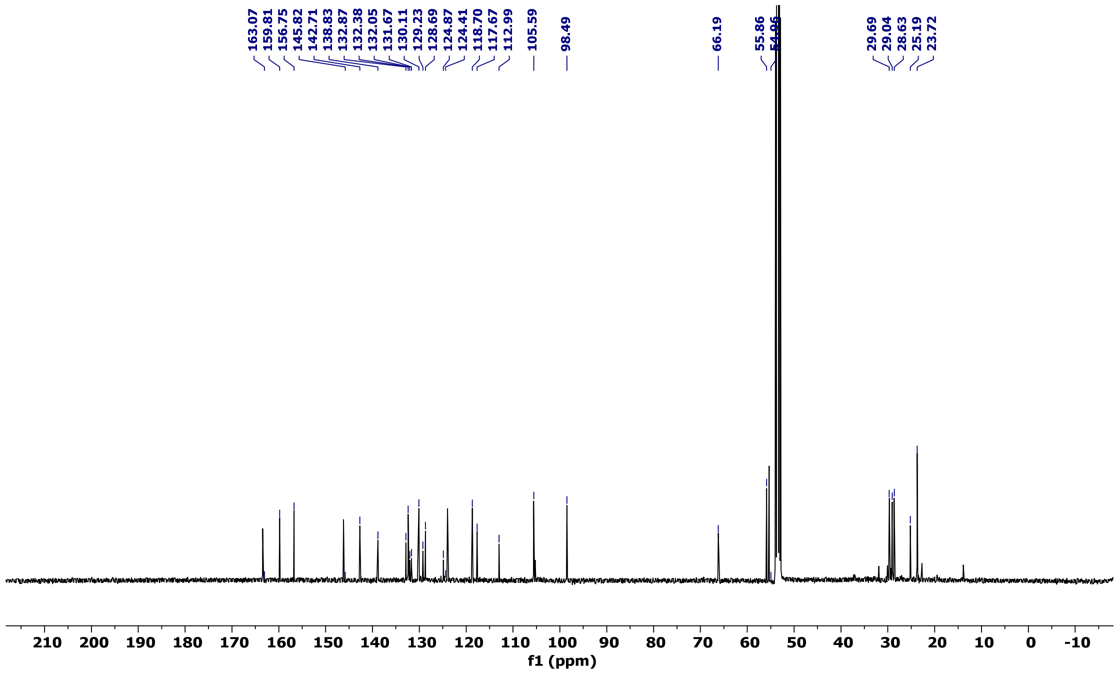

^{1}H NMR (400 MHz, THF-d8, 293 K, ppm) of **PMIP-OH**

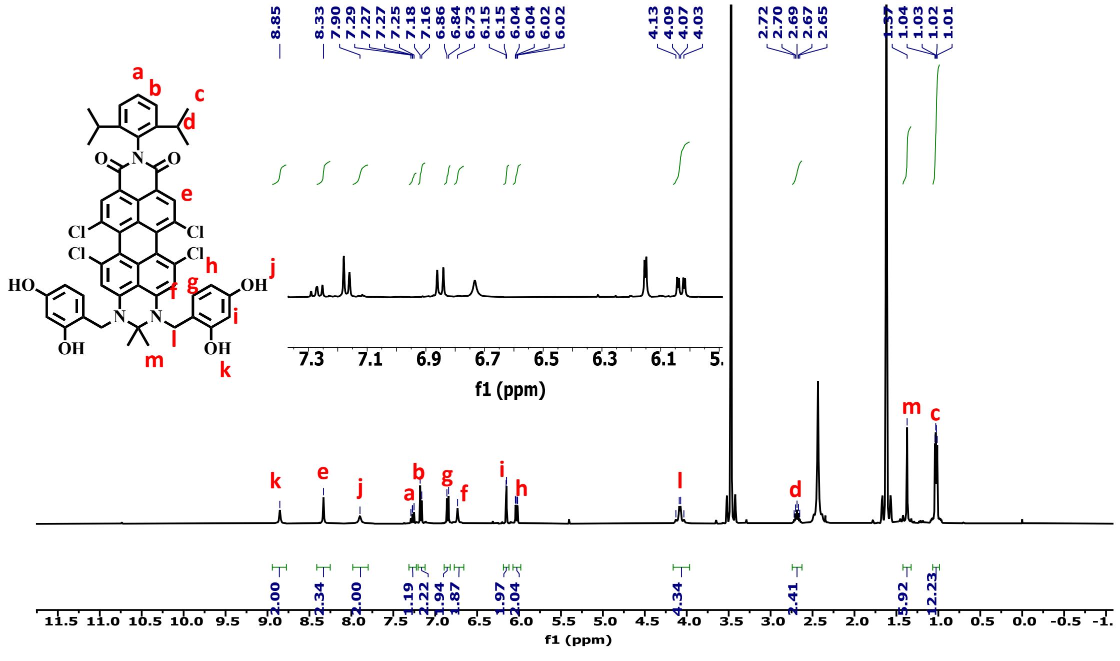

^{1}H NMR (400 MHz, CD_2Cl_2, 293 K, ppm) of **4**

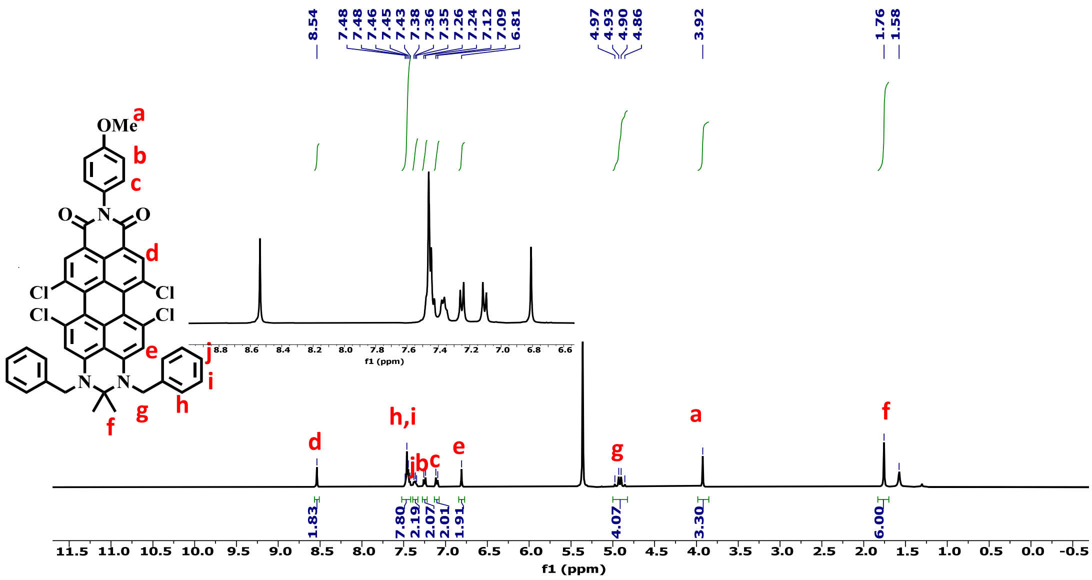

^{13}C NMR (400 MHz, CD_2Cl_2, 293 K, ppm) of **4**

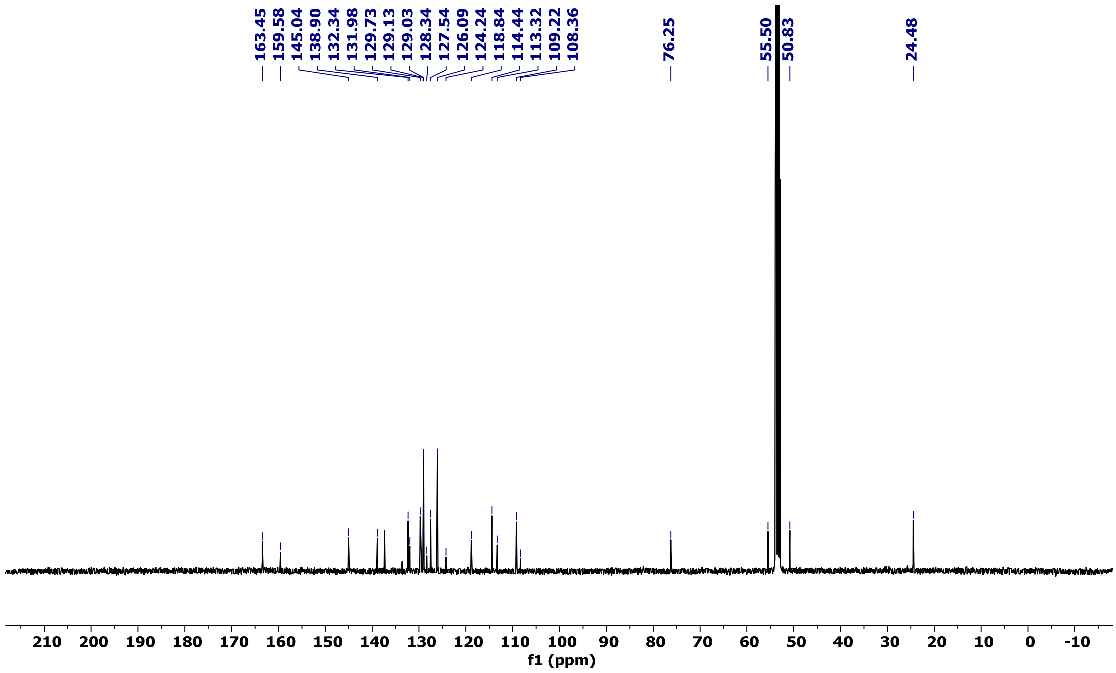

^{1}H NMR (400 MHz, THF-d8, 293 K, ppm) of **6**

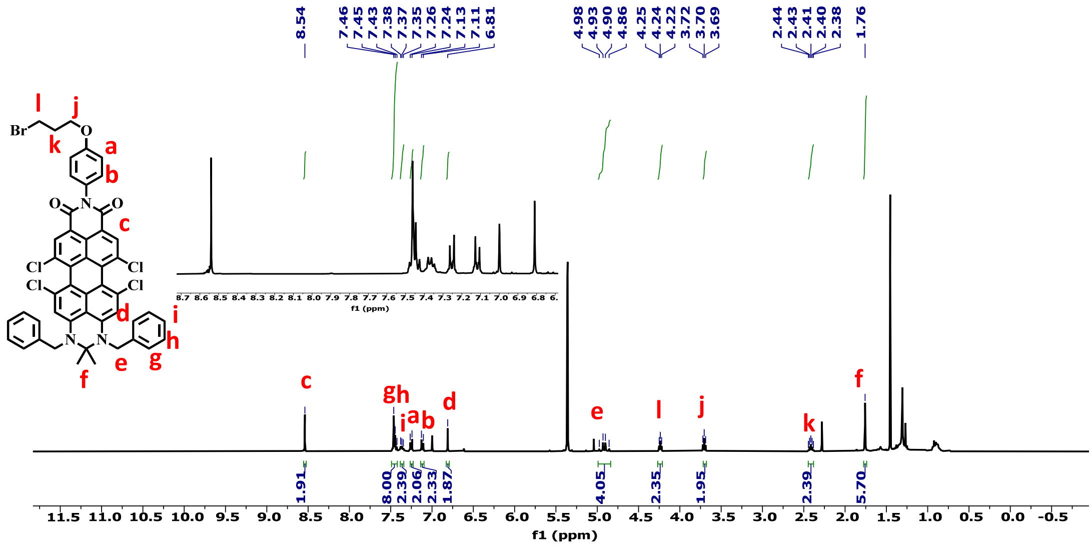

^{13}C NMR (101 MHz, CD_2Cl_2, 293 K, ppm) of **6**

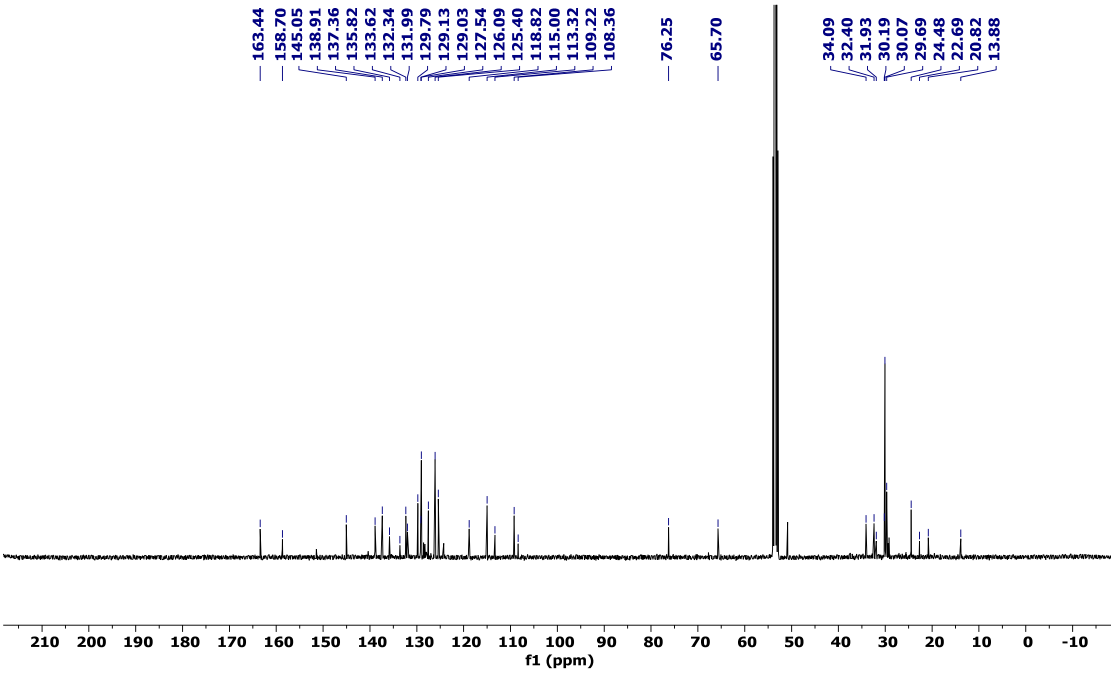

^{1}H NMR (400 MHz, CD_2Cl_2, 293 K, ppm) of **PMIP-N_3**

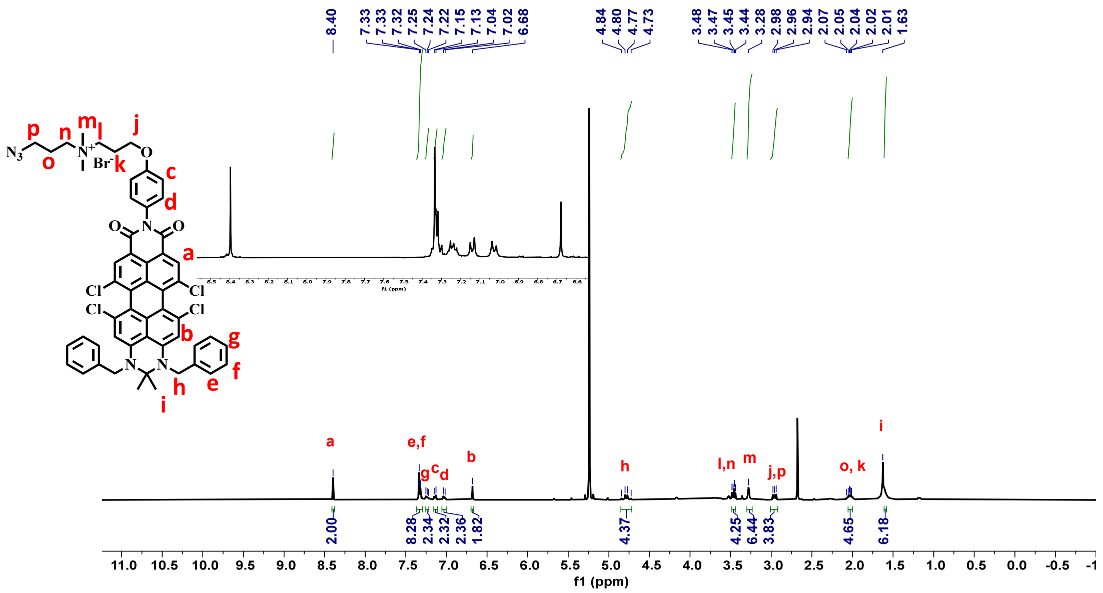

13. References

1. Liu, X., Chen, S.-Y., Chen, Q., Yao, X., Gelléri, M., Ritz, S., Kumar, S., Cremer, C., Landfester, K., Müllen, K., Parekh, S. H., Narita, A., Bonn M. Nanographenes: Ultrastable, Switchable, and Bright Probes for Super-Resolution Microscopy. *Angew. Chemie Int. Ed.* **2020**, *59*, 496–502.
2. Tanaka, T., Yamagami, T., Nogami, T., Minami, H. & Okubo, M. Preparation of hemispherical polystyrene particles utilizing the solvent evaporation method in aqueous dispersed systems. *Polym. J.* **2012**, *44*, 1112–1116.
3. Ovesný, M., Křížek, P., Borkovec, J., Švindrych, Z. & Hagen, G. M. ThunderSTORM: A comprehensive ImageJ plug-in for PALM and STORM data analysis and super-resolution imaging. *Bioinformatics* **2014**, *30*, 2389–2390.
4. Dempsey, G., Vaughan, J., Chen, K., Bates, M., Zhuang, X. Evaluation of fluorophores for optimal performance in localization-based super-resolution imaging. *Nat. Methods* **2011**, *8*, 1027–1036.
5. Schindelin, J., Arganda-Carreras, I., Frise, E., Kaynig, V., Longair, M., Pietzsch, T., Preibisch, S., Rueden, C., Saalfeld, S., Schmid, B., Tinevez, J-Y., White, D. J., Hartenstein, V., Eliceiri, K., Tomancak, P., Cardona, A. Fiji: an open-source platform for biological-image analysis. *Nat. Methods* **2012**, *9*, 676–682.
